# Rifampicin reduces advanced glycation end products and activates DAF-16 to increase lifespan in *Caenorhabditis elegans*

**DOI:** 10.1111/acel.12327

**Published:** 2015-02-26

**Authors:** Sandeep Golegaonkar, Syed S Tabrez, Awadhesh Pandit, Shalini Sethurathinam, Mashanipalya G Jagadeeshaprasad, Sneha Bansode, Srinivasa-Gopalan Sampathkumar, Mahesh J Kulkarni, Arnab Mukhopadhyay

**Affiliations:** 1 Division of Biochemical Sciences, CSIR-National Chemical LaboratoryPune, 411008, India; 2 Molecular Aging Laboratory, National Institute of ImmunologyAruna Asaf Ali Marg, New Delhi, 110067, India; 3 Laboratory of Chemical Glycobiology, National Institute of ImmunologyAruna Asaf Ali Marg, New Delhi, 110067, India

**Keywords:** advanced glycation end products, aging, *Caenorhabditis elegans*, DAF-16, glycation, lifespan, rifampicin

## Abstract

Advanced glycation end products (AGEs) are formed when glucose reacts nonenzymatically with proteins; these modifications are implicated in aging and pathogenesis of many age-related diseases including type II diabetes, atherosclerosis, and neurodegenerative disorders. Thus, pharmaceutical interventions that can reduce AGEs may delay age-onset diseases and extend lifespan. Using LC-MS^E^, we show that rifampicin (RIF) reduces glycation of important cellular proteins *in vivo* and consequently increases lifespan in *Caenorhabditis elegans* by up to 60%. RIF analog rifamycin SV (RSV) possesses similar properties, while rifaximin (RMN) lacks antiglycation activity and therefore fails to affect lifespan positively. The efficacy of RIF and RSV as potent antiglycating agents may be attributed to the presence of a *p*-dihydroxyl moiety that can potentially undergo spontaneous oxidation to yield highly reactive *p*-quinone structures, a feature absent in RMN. We also show that supplementing rifampicin late in adulthood is sufficient to increase lifespan. For its effect on longevity, rifampicin requires DAF-18 (nematode PTEN) as well as JNK-1 and activates DAF-16, the FOXO homolog. Interestingly, the drug treatment modulates transcription of a different subset of DAF-16 target genes, those not controlled by the conserved Insulin-IGF-1-like signaling pathway. RIF failed to increase the lifespan of *daf-16* null mutant despite reducing glycation, showing thereby that DAF-16 may not directly affect AGE formation. Together, our data suggest that the dual ability to reduce glycation *in vivo* and activate prolongevity processes through DAF-16 makes RIF and RSV effective lifespan-extending interventions.

## Introduction

Aging is an inescapable process in all living beings. Although most animals exhibit signs of age-related decline in body functions, some interestingly show negligible visible signs of senescence (e.g., some tortoises, sea anemones, lobsters). One of the hallmarks of aging is the accumulation of altered proteins (Hipkiss, 2006). With advancing age, cellular homeostatic processes that restrict damage caused by altered proteins decline in their efficiency, leading to various age-related pathologies (Thornalley, 2008). Importantly, modern lifestyle also contributes immensely to this process of disease development. One such posttranslational alteration of protein, which is at the center of many age-related diseases including type II diabetes, atherosclerosis, renal disorders, Alzheimer's disease, and rheumatoid arthritis, is the formation of advanced glycation end products (AGEs) (Thornalley, 2008; Luevano-Contreras & Chapman-Novakofski). AGEs are a complex and heterogeneous group of molecules formed via a series of parallel and sequential nonenzymatic reactions involving glucose or glucose-derived products and amino groups of proteins, lipids, or DNA (Singh *et al*., 2001; Ulrich & Cerami, 2001; Ahmed, 2005; Luevano-Contreras & Chapman-Novakofski, 2010). The carbonyl group of a sugar reacts with a protein to form an unstable aldimine intermediate called Schiff's base (Fig. S1A). The Schiff's base may undergo an Amadori rearrangement to form a stable 1-amino-1-deoxyfructose derivative with a stable ketoamine linkage, commonly known as Amadori product. The formation of Amadori product marks the reversible phase of glycation that is followed by irreversible oxidation, dehydration, condensation, fragmentation, or cyclization leading to the formation of AGEs such as pentosidine, *N-*ε*-*carboxymethyllysine (CML), and pyraline (Singh *et al*., 2001; Ulrich & Cerami, 2001; Ahmed, 2005). Additionally, auto-oxidation of glucose by the Wolff pathway or of the Schiff's base by the Namiki pathway generates highly reactive dicarbonyl or oxoaldehyde molecules such as glyoxal, methylglyoxal, or 3-deoxyglucosone (3-DG). The dicarbonyl intermediates in turn give rise to AGEs such as pentosidine, CML as well as glyoxal-lysine dimer (GOLD), or methylglyoxal-lysine dimer (MOLD) through additional cross-linking (Khalifah *et al*., 1999; Singh *et al*., 2001; Ulrich & Cerami, 2001; Ahmed, 2005).

Methylglyoxal is mainly formed during the process of glycolysis; either enzymatically by methylglyoxal synthase, cytochrome 2E1, and semicarbazide-sensitive amine oxidase or nonenzymatically by spontaneous degradation of dihydroxyacetone phosphate and glyceraldehyde-3-phosphate. Under euglycemic conditions, the glyoxalase system breaks down methylglyoxal and converts it to D-lactate (Rabbani & Thornalley, 2011; Xue *et al*., 2011). However, this cellular detoxification machinery loses efficacy with increasing age. As a result, AGEs build up in different tissues as an organism gets older (Semba *et al*., 2010). AGE modifications often lead to abnormal protein function, oxidative stress, and inflammation; thus, they are closely associated with deteriorating cellular functions during aging and in pathologies of several age-related diseases (Luevano-Contreras & Chapman-Novakofski, 2010; Xue *et al*., 2011). In *C. elegans*, a powerful genetic model that has emerged as an effective system to study aging, glyoxalase I activity is markedly reduced with age that leads to increased oxidative stress. Interestingly, overexpression of the gene can decrease methylglyoxal-induced mitochondrial protein modifications and increase lifespan (Morcos *et al*., 2008). Thus, pharmaceutical interventions that can suppress AGE formation may be an effective way to increase lifespan and health span (Semba *et al*., 2010). In this study, we show that rifampicin (RIF), a potent glycation inhibitor (Golegaonkar *et al*., 2010), dramatically increases lifespan (up to 60%) as well as improve health of *C. elegans* by preventing AGE modifications of important cellular proteins. This is by far one of the largest increases in lifespan obtained using a pharmaceutical reagent. Apart from possessing antiglycating activity, RIF activates the FOXO transcription factor DAF-16, possibly through JNK pathway, to modulate transcription of a unique set of target genes, those that are not controlled by the Insulin-IGF-1 signaling pathway. Rifaximin (RMN), a rifampicin analog that lacks antiglycating activity *in vitro* and *in vivo*, but possesses the ability to activate DAF-16 fails to extend lifespan. Together, our study shows that the dual ability of RIF to reduce glycation as well as activate DAF-16 makes it a potent lifespan-extending intervention.

## Results

### Rifampicin and rifamycin SV dramatically increase lifespan

**Fig 1:**
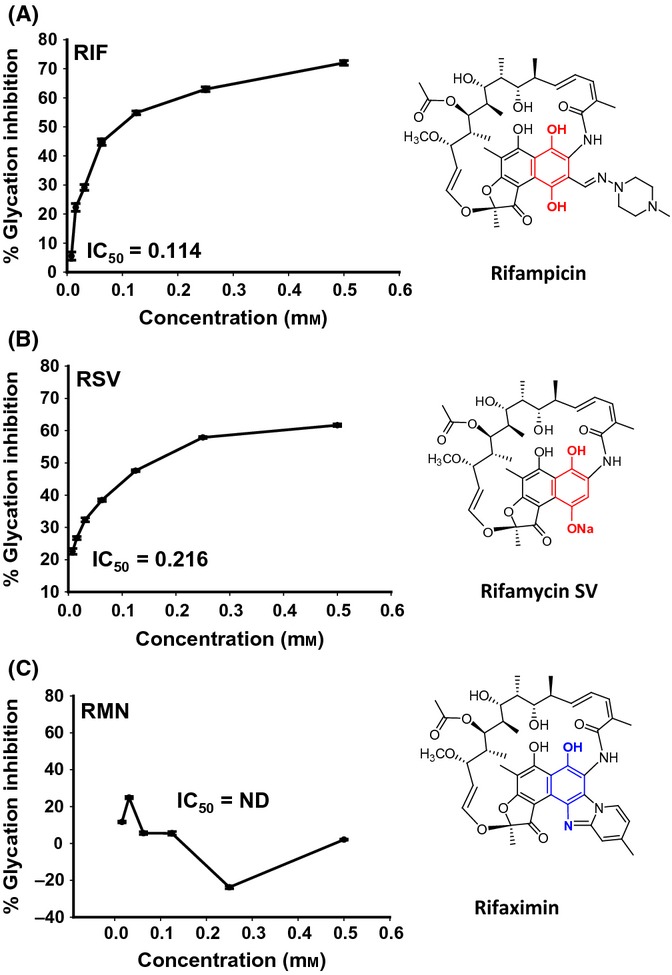
RIF treatment prevents Schiff's base formation *in vitro*. Insulin (100 μg) was incubated with 500 mm glucose along with increasing concentrations of RIF (A), RSV (B), or RMN (C). The *in vitro* Schiff's base formation was measured using MALDI-TOF-TOF. Percentage inhibition of glycation was calculated over the concentration range indicated on *x*-axis and IC_50_ determined. The chemical structures of the compounds are shown in the right panels. Note that RIF and RSV possess the *para*-dihydroxynaphthyl moiety (highlighted in red), whereas RMN possesses a *para*-aminophenyl moiety in a fused ring system (highlighted blue). ND—not determined.

**Fig 2:**
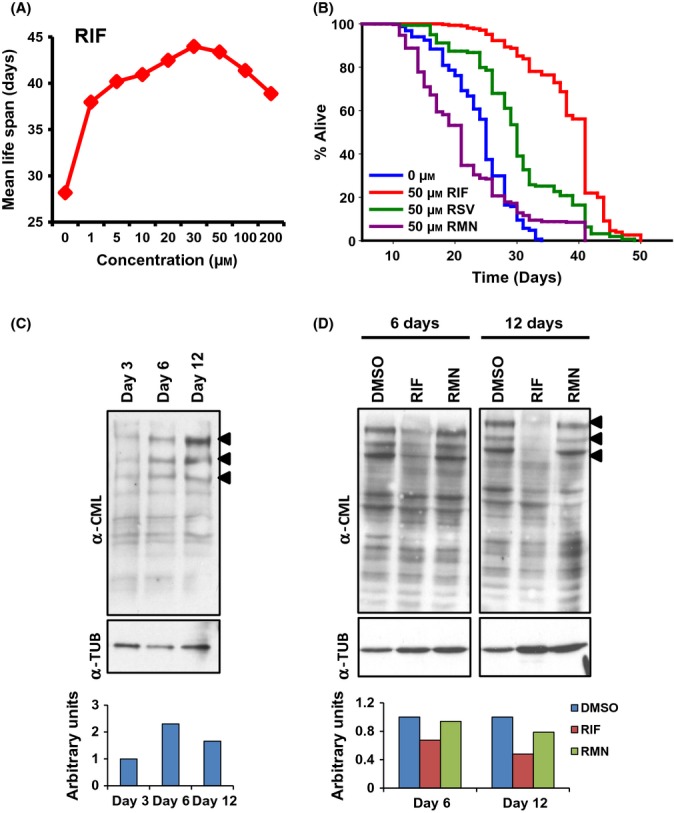
RIF treatment reduces glycation *in vivo* and positively affects *C. elegans* lifespan. (A) RIF supplementation extended lifespan of wild-type (WT) worms grown on heat-killed *E. coli*OP50, in a concentration-dependent manner. Lifespan analysis was performed in the presence of increasing concentrations of RIF, and mean lifespans were plotted. Only DMSO was added in the control experiment (0 μmRIF). (B) RIF and its analog RSV increased lifespan significantly, while RMN had no positive effect. (C) Levels of the AGE carboxymethyl lysine (CML) increased with progressing age, as determined by Western blot analysis using α-CML antibody. WT were worms collected on day 3, 6, or 12 of adulthood. Arrowhead indicates the major bands that changed in expression with age. (D) RIF treatment, but not DMSO or RMN, reduced CML levels as determined by Western blot in WT worms collected on day 6 or 12. The α-tubulin (α-TUB) levels were used for normalization. Relative quantification of band intensity is provided below each panel.

Repositioning of known drugs for novel applications is an emerging strategy in the area of drug discovery. In our previous study using an *in vitro* mass spectrometry-based screen, the antibiotic rifampicin (RIF) was identified as a more potent glycation inhibitor than aminoguanidine (AMG), a well-known antiglycating agent (Golegaonkar *et al*., 2010). The higher efficacy of rifampicin as a glycation inhibitor was also evidenced in case of AGE-induced corneal stiffening (Brummer *et al*., 2011). Similar to RIF (apparent IC_50_ 0.114 mM), its analog rifamycin SV (RSV) also has similar glycation inhibition efficiency (apparent IC_50_ 0.216 mM) (Figs1A,B, S1B). On the other hand, another analog, rifaximin (RMN), was found to possess low *in vitro* activity as a glycation inhibitor (Figs1C, S1B). The differential activity of RIF, RSV, and RMN could be attributed to their chemical structures. All the three compounds possess macrocyclic lactam structure containing naphthyl moiety fused with a cyclopentanone ring as a common feature. However, only RIF and RSV possess the *para*-dihydroxynaphthyl moiety (highlighted in red in Fig.1A,B), while RMN has a *para*-aminophenyl moiety fused in a ring system (highlighted in blue in Fig.1C). As AGE accumulation with increasing age is linked to diseases and reduced longevity (Luevano-Contreras & Chapman-Novakofski, 2010), we asked whether glycation inhibitors RIF and RSV have any positive effect on lifespan. Using a modified *C. elegans* liquid lifespan assay with live *E. coli* OP50 (see Experimental procedures), we observed that RIF significantly increased lifespan of wild-type worms (Fig. S2A, details in Table s3). As *E. coli* OP50, which is used to feed *C. elegans* in the laboratory, can act as an opportunistic pathogen in older worms, antibiotics such as RIF may increase lifespan by simply killing the bacteria. To rule out this possibility, we repeated the above experiment using heat-killed bacteria. We found that RIF was still able to increase lifespan in a concentration-dependent manner (Fig.2A, Table s3) ruling out two scenarios: (i) Its effect on lifespan was due to bactericidal properties and (ii) bacteria metabolizing the drug to produce an unrelated product that indirectly affected lifespan. Additionally, antibiotic kanamycin did not have similar effect on lifespan (Fig. S2B). Therefore, we continued all our experiments using heat-killed bacteria and used 50 μm RIF. Next, we analyzed RSV and RMN for their effect on lifespan. While RSV increased lifespan significantly like RIF (Mean Lifespan ± Standard Error Mean on 0 μm RIF is 24.89 ± 0.28, while on 50 μm RIF is 39.57 ± 0.29 and 50 μm RSV is 30.09 ± 0.61, *P *≤* *0.0001 in all cases; Fig.2B), RMN treatment had no positive effect on lifespan (MLS ± SEM on 50 μm RMN is 21.48 ± 0.46 *P *≤* *0.0001) (Fig.2B, Table s3). In fact, RMN significantly reduced lifespan. Finally, treatment with RIF also enhanced two parameters that are representative of better health in worms, that is, decreased accumulation of autofluorescence in the gut (Fig. S2C) and delayed onset of proteotoxicity (Fig. S2D). These experiments suggested that the increased lifespan and health on RIF and RSV treatment may be due to their potent antiglycation activity *in vivo*.

### Rifampicin reduces AGE accumulation *in vivo*

Increased AGE formation associated with progressing age is linked to many diseases including atherosclerosis and neurodegenerative disorders (Nedic *et al*., 2013). Using a monoclonal antibody that detects carboxymethyl lysine (CML), we performed Western blot analysis with proteins extracted from 3-, 6-, or 12-day-old adult wild-type worms. We found that accumulation of CML increased with progressing age in worms (Fig.2C). As expected from *in vitro* glycation assays and lifespan analysis, treatment with RIF decreased CML formation considerably in 6- and 12-day-old worms (Fig.2D). However, RMN treatment had much lesser effect on CML formation on different days of adulthood, compared to RIF (Fig.2D). These experiments show that AGE formation increases with age in *C. elegans* and RIF can act as a glycation inhibitor *in vitro* and *in vivo* to affect lifespan positively. Importantly, the property of RIF to increase lifespan can be linked to its antiglycating property.

Methyl glyoxal (MG) is a highly reactive dicarbonyl that reacts with epsilon amino groups of lysine and sulfhydryl groups of proteins leading to the formation of AGEs. The levels of glyoxalase decline with age leading to increased AGE modification of proteins; AGE levels, including CML, also rise when glyoxalase is knocked down (Morcos *et al*., 2008). The *glod-4* mutant lived significantly shorter than the WT (Fig. S3A), possibly due to the increased accumulation of AGEs. Interestingly, RIF was able to decrease CML modification of proteins in *glod-4(gk189)* (Fig. S3B) and consequently increased lifespan of the mutant worms (MLS ± SEM on 0 μm RIF is 22.78 ± 0.23, while on 50 μm RIF is 36.88 ± 0.31, *P *≤* *0.0001) (Fig. S3C) similar to WT (shown above in Fig.2B).

Next, we used LC-MS^E^, a data-independent acquisition (DIA) strategy to identify *in vivo* AGE-modified proteins. We used the *glod-4(gk189)* strain as we thought that it may have relatively higher total AGE levels that will be easier to detect. LC-MS^E^ is a unique approach wherein all eluted peptides are fragmented and the fragment ions are time-aligned with the retention time of the peptides (Silva *et al*., 2006). This method allows analysis of low abundance posttranslationally modified peptides (Blackburn & Goshe, 2009; Xie *et al*., 2009). A total of 49 AGE-modified proteins were detected in DMSO- and RIF-treated samples. In the DMSO-treated samples, 69 peptides carrying 89 AGE modifications were detected. However, in the RIF-treated samples, only 32 peptides were observed to carry 42 AGE modifications (Table1). The decrease in AGE modifications on proteins was not directly correlated to the relative changes in protein expression on RIF treatment (Table s4). We found that the beta subunit of the mitochondrial ATP synthase possessed the most number of modifications that were dramatically reduced on RIF treatment. VIT-2 as well as VIT-6, a vitellogenin that is reported to be heavily carbonylated in worms (Nakamura *et al*., 1999), also had many modifications that showed reduction on RIF treatment. Interestingly, modifications on several mitochondrial proteins including ATP synthase subunit beta, aconitate hydratase, isoform D of ATP synthase subunit alpha, dihydrolipoyl dehydrogenase, methylmalonate semialdehyde dehydrogenase, and chaperonin Hsp 60 as well as two fatty acid and retinol binding proteins also decreased on RIF treatment. Marked decrease in glycation was also reported in mitochondrial proteins when glyoxalase I was overexpressed in *C. elegans* (Morcos *et al*., 2008). Apart from metabolic proteins, RIF treatment resulted in a decrease in glycation on muscle-specific proteins such as paramyosin, tropomyosin, and myosin heavy and light chain as well as structural proteins such as actin and tubulin. Modifications on these important proteins may disrupt normal cellular functions leading to aging. RIF may help in retaining proper functioning of these proteins and thereby affect lifespan positively.

**Table 1:**
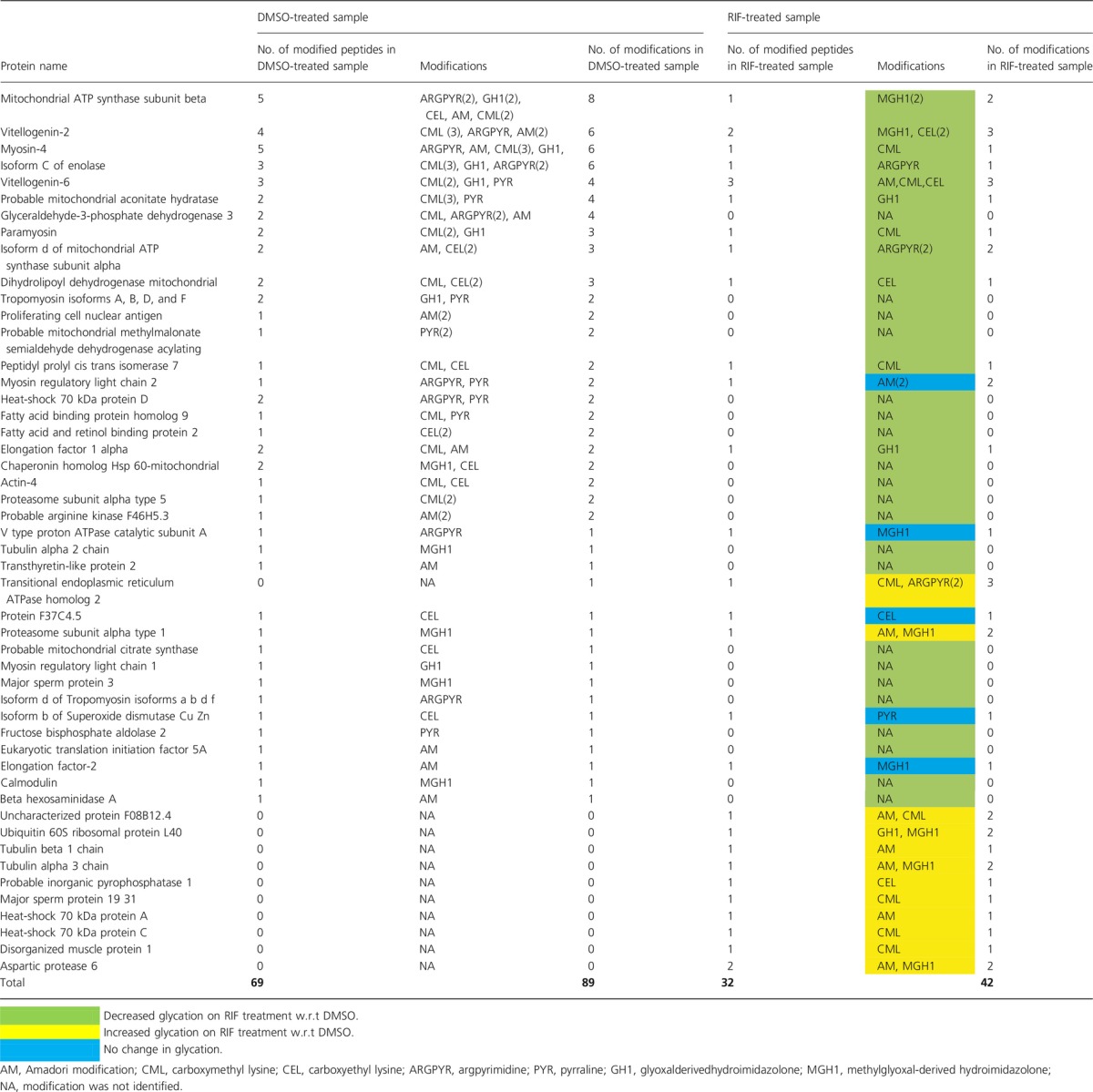
List of proteins detected by LC-MS^E^, along with their AGE modifications, following treatment with RIF

### Rifampicin activates FOXO homolog, DAF-16

**Fig 3:**
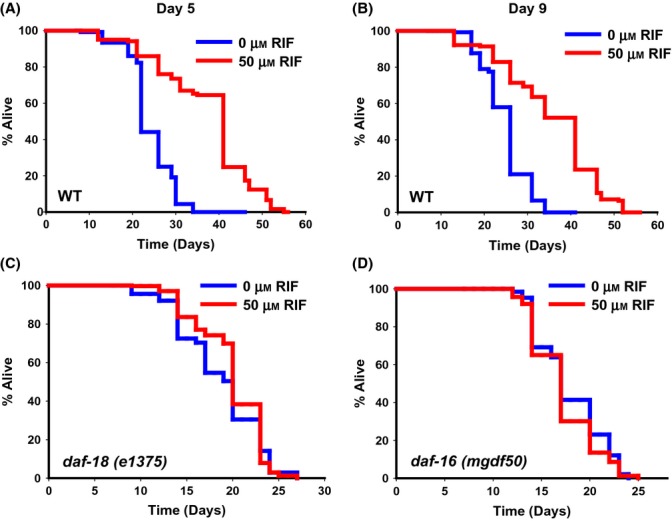
RIF requires components of the IIS pathway to increase lifespan. Supplementation of RIF on day 5 (A) or day 9 (B) of adulthood increased lifespan of wild-type worms to varying extent. RIF failed to extend lifespan of (C) *daf-18(e1375)*, a mutant in the worm PTEN homolog or (D) *daf-16(mgdf50)*, a mutant in the worm FOXO gene.

The Insulin-IGF-1 signaling (IIS) pathway in *C. elegans* and other organisms is one of the most important regulators of aging (Kenyon, 2010). Mutations in the components of IIS increase lifespan across the animal kingdom. Interestingly, genetic experiments in worms have revealed distinct temporal requirements for the longevity pathways to affect lifespan (Dillin *et al*., 2002). While IIS pathway knockdown starting on the first day of adulthood or even later is sufficient to extend lifespan, mitochondrial genes have to be knocked down during larval stages for increased longevity (Dillin *et al*., 2002). Similar to IIS pathway knockdown, RIF supplementation starting at day 1, 5, or even at day 9 of adulthood increased lifespan significantly (Figs2B and 3A,B, Table s3). This highlights the potency of RIF to act as a single-dose late-life longevity-extending intervention.

As RIF has a temporal requirement similar to the IIS pathway mutants for regulating longevity, we studied its relation to this pathway. Mutations in the Insulin-IGF-1-like receptor, *daf-2*, reduces signaling through this pathway and increases lifespan (Kenyon, 2010). Mutation in the lipid phosphatase DAF-18 (homolog of mammalian PTEN) activates the IIS and suppresses the long lifespan of *daf-2* mutants (Ogg & Ruvkun, 1998; Mihaylova *et al*., 1999). We observed that RIF failed to increase the lifespan in *daf-18(e1375)* mutant (MLS ± SEM on 0 μm RIF is 18.60 ± 0.26, while on 50 μm RIF is 19.84 ± 0.21, *P* = 0.0739; Fig.3C, Table s3), suggesting that DAF-18 is required for RIF-induced lifespan extension. The increased lifespan of a *daf-2* mutant is completely dependent on the FOXO transcription factor DAF-16 (Lin *et al*., 1997; Ogg *et al*., 1997). Further, the RIF-mediated lifespan extension also requires DAF-16 as the drug failed to increase lifespan in *daf-16(mgdf50)*, a null mutant (MLS ± SEM on 0 μm RIF is 17.80 ± 0.25, while on 50 μm RIF is 17.11 ± 0.25, *P* = 0.122) (Fig.3D, Table s3). These experiments suggested that RIF treatment may genetically interact with the IIS pathway components and activate DAF-16.

**Fig 4:**
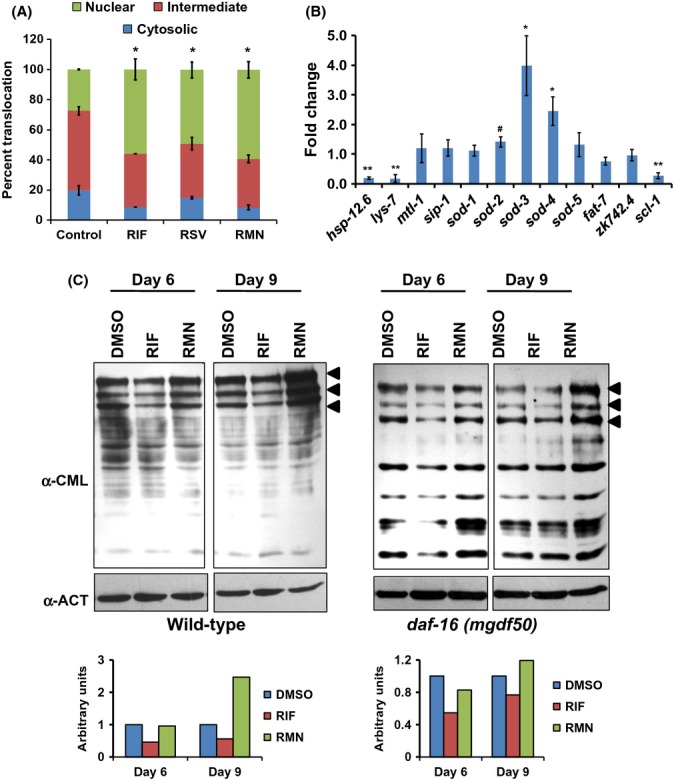
RIF treatment activates DAF-16 in WT worms, but *daf-16* is not required for its antiglycating effects. (A) RIF, RSV, and RMN significantly increased DAF-16 nuclear translocation in TJ356, a DAF-16::GFP-expressing strain. Worms treated with equivalent concentration of DMSO was taken as control. (B) Differential regulation of a set of genes, including DAF-16 targets, by RIF as compared to DMSO treatment, determined by quantitative real-time PCR. **P ≤* *0.001, *P ≤* *0.01, ^#^*P *≤* *0.05 (C) RIF treatment decreased CML accumulation in WT (left panel) as well as in *daf-16(mgdf50)*(right panel) as determined by Western blot analysis. Relative quantification of band intensity is provided below each panel. The α-Actin (α-ACT) levels were used for normalization.

DAF-16 shuttles between the nucleus and cytoplasm; it translocates into the nucleus in response to low Insulin signaling or under stress. Activation of DAF-16 by RIF was supported by the enhancement of DAF-16::GFP protein (Henderson & Johnson, 2001) translocation into the nucleus in the presence of the drug (Fig.4A) and consequently, upregulation of *sod-3*, its direct target (Oh *et al*., 2006) (Fig.4B). Similar to RIF, RSV was also able to translocate DAF-16 into the nucleus (Fig.4A). Together, activation of DAF-16 is an additional mechanism of RIF-mediated lifespan extension, apart from its antiglycating property. Incidentally, RMN that lacked the glycation inhibition property also affected DAF-16 translocation (Fig.4A). Thus, our data also indicate that a combination of potent antiglycating activity and positive regulation of DAF-16 is required for extending lifespan as observed on RIF treatment. Simply possessing DAF-16-activating property may not be sufficient to increase lifespan, as observed for RMN.

There existed a possibility that activation of DAF-16 leads to the upregulation of an AGE detoxification system, resulting in increased AGE disposal and a consequential increase in lifespan. To evaluate this possibility, we grew *daf-16(mgdf50)* in the presence of RIF and measured the CML levels on days 6 and 9 of adulthood. We found that in *daf-16(mgdf50),* RIF was still able to decrease CML formation (Fig.4C). Although we cannot rule out the possibility that other AGEs are unaffected or that small suppression of CML can have a dramatic effect on lifespan, our data suggest that DAF-16 may have other functions in lifespan regulation downstream of RIF, apart from detoxifying and disposing AGE-modified proteins.

### Rifampicin may activate DAF-16 through JNK-1

**Fig 5:**
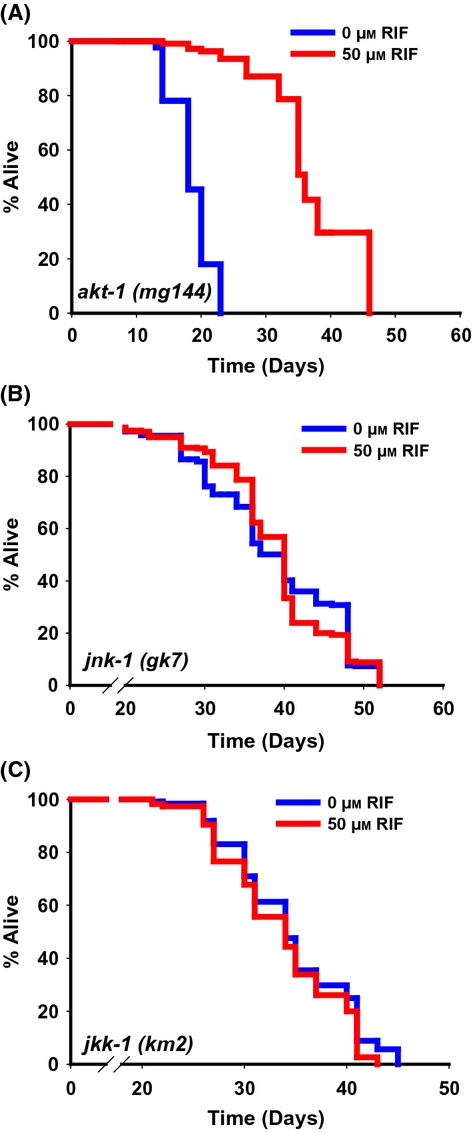
RIF requires components of the JNK pathway to increase lifespan. (A) RIF supplementation increased lifespan of an activated allele of *akt-1*, *akt-1(mg144)*. RIF failed to extend lifespan of (B) JNK-1 deletion mutant *jnk-1(gk7)* and (C) JNK-1 upstream kinase JKK-1 deletion mutant *jkk-1(km2)*, to a significant extent.

Next, we asked whether RIF treatment reduces signals through the well-conserved Ser-Thr kinases, PDK-1 and AKT-1, that are downstream to the regulation by DAF-18, but upstream of DAF-16. Activation of these kinases leads to phosphorylation and exclusion of DAF-16 from the nucleus, while mutations in these kinase genes lead to prolonged lifespan in a DAF-16-dependent manner (Paradis & Ruvkun, 1998; Paradis *et al*., 1999). We found that in the activated alleles *pdk-1(mg142)* (MLS ± SEM on 0 μm RIF on 29.18 ± 0.40, while on 50 μm RIF is 35.99 ± 0.47, *P *≤* *0.0001, Fig. S4) and *akt-1(mg144)* (MLS ± SEM on 0 μm RIF is 18.55 ± 0.26, while on 50 μm RIF is 36.96 ± 0.70, *P *≤ 0.0001, Fig.5A), RIF could still increase lifespan, indicating that it may utilize a separate signaling pathway, bypassing PDK-1. As RIF treatment may activate an oxidative stress response pathway as in mammals (Sodhi *et al*., 1997), we speculated a role of JNK-1, a known DAF-16 and FOXO activator (Oh *et al*., 2005). In line with this supposition, RIF failed to considerably increase the lifespan of *jnk-1(gk7)* (MLS ± SEM on 0 μm RIF is 39.52 ± 0.61, while on 50 μm RIF is 39.14 ± 0.51, *P* = 0.6307; Fig.5B). In fact, the upstream kinase for JNK-1, JKK-1 (Oh *et al*., 2005), is also involved in this process as in *jkk-1(km2)*, RIF was not able to increase lifespan (MLS ± SEM on 0 μm RIF is 33.38 ± 0.43, while on 50 μm RIF is 33.96 ± 0.47, *P* = 0.3088; Fig.5C). Together, these genetic data suggest that RIF requires some specific components of the IIS pathway and may utilize JNK pathway to activate DAF-16 and enhance lifespan. Further experimentation is mandated in dissecting the role of JNK pathway in RIF-mediated lifespan extension.

### Rifampicin modulates expression of a different subset of DAF-16 target genes

**Fig 6:**
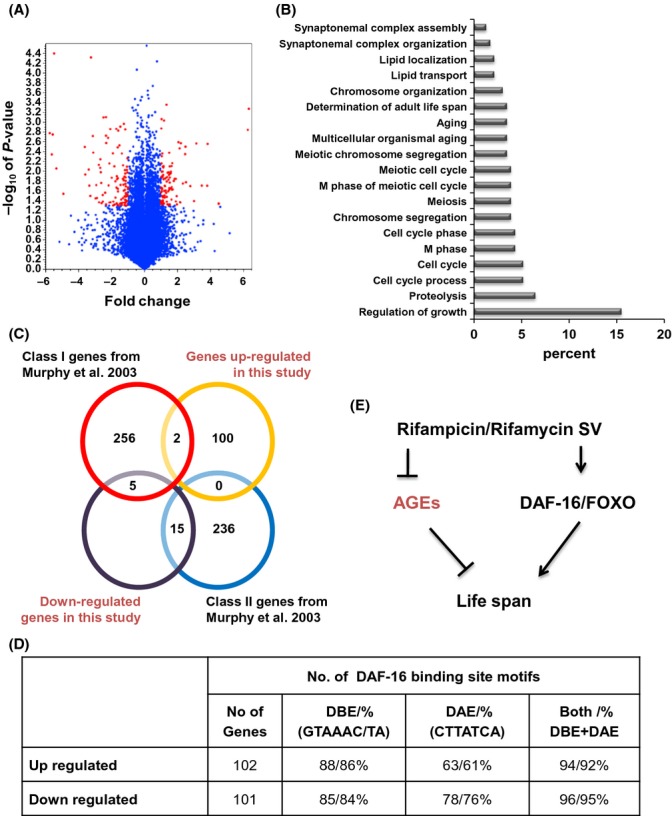
RIF regulates a different subset of DAF-16 target genes, not controlled by the IIS pathway. (A) A volcano plot showing genes (red dots) that significantly change expression on RIF treatment. Blue dots represent genes that do not have significant expression changes. (B) GO term analysis of genes upregulated significantly on RIF treatment. (C) Overlap of genes identified by microarray analysis following RIF treatment (this study) with genes reported by Murphy *et al*. (2003) as targets of DAF-16 (under the control of IIS pathway). Class I genes are upregulated, while class II genes are downregulated in *daf-2(e1370)* mutant, in a *daf-16-*dependent manner. (D) Analysis of 5-kb upstream region of genes whose expression changed on RIF treatment revealed significant enrichment of DAF-16 binding element (DBE) and associated element (DAE) (*P* value is 8.28e-10 by hypergeometric test). (E) A model for lifespan extension by RIF and RSV. These compounds are potent glycation inhibitors that have DAF-16 activating function; both activities are required for increasing lifespan in *C. elegans*.

To further understand the mechanism of RIF action, we performed a comparative microarray analysis which revealed that 102 genes are significantly upregulated, while 104 genes were downregulated by at least two fold (*P *<* *0.05) upon RIF treatment as compared to DMSO (Fig.6A; Tables S1 and S2). While most of these genes are uncharacterized/unannotated, GO term analysis revealed that genes involved in cell cycle regulation, proteolysis, and organismal aging were enriched in the upregulated gene list (Fig.6B). On the other hand, many genes involved in transcriptional regulation were downregulated (Fig. S5A). We also found genes that possibly function in countering AGEs to be downregulated (e.g., lysozymes, saposin-like proteins, peptidases); products of these genes may not be required due to the low intrinsic AGEs upon RIF treatment. Alternatively, the downregulation of lectins, saposins, and lysozymes may indicate that the worms may not be mounting innate immune response due to the presence of antibiotic in the media. However, two genes that have putative function as ubiquitin ligase and ubiquitin-conjugating enzyme were upregulated possibly to facilitate removal of nonfunctional proteins. Also, Vitellogenin genes (*vit-3*, *vit-4* and *vit-5*) were found to be downregulated significantly. These proteins accumulate in the intestine following reproductive development and are subjected to oxidative damage leading to toxicity. So, lower levels of these proteins may promote longevity as shown earlier (Murphy *et al*., 2003; Van Nostrand *et al*., 2013). Additionally, genes involved in xenobiotic detoxification (e.g., cytochrome P450, UDP-glucuronosyltransferase, P-glycoprotein-related proteins) were upregulated (Table s1). The latter group supports increased longevity as recently reported (Shore & Ruvkun, 2013) and may also be involved in detoxification of RIF. RIF treatment thus brings about a profound change in the *C. elegans* transcriptome that may also be responsible for the increased longevity.

A previous microarray study has revealed several hundred genes that are up- (Class 1) or downregulated (Class II) in a *daf-2* mutant, dependent on DAF-16 (Murphy *et al*., 2003). We observed that although a few Class I DAF-16 target genes had increased expression on RIF treatment, some others of the same class were downregulated. Quantitative RT–PCR confirmed this as *sod-3* was significantly upregulated in the presence of RIF, while *scl-1* and *hsp-12.6* were downregulated (Fig.3B). Although both RIF and a mutation in *daf-2* seem to activate DAF-16, there was only 2% overlap with Class I genes and 6% commonality with Class II genes between the two regimes, highlighting a separate activation profile of RIF (Fig.6C). However, 92–95% of the promoters (5 kb upstream) of genes that are up- or downregulated following RIF treatment had consensus DAF-16 binding (GTAAAC/TAA) as well as DAF-16-associated (CTTATCA) elements (Fig.6D, Tables S1 and S2) (Murphy *et al*., 2003), compared to 78% as would be expected by chance (*P* value is 8.28e-10 by hypergeometric test considering 20447 coding genes in WBcel235) (Kenyon & Murphy, 2006). Using quantitative real-time PCR, we checked the expression of 10 genes following RIF treatment and found seven of them to change in a *daf-16*-dependent manner (Fig. S5B). Thus, we conclude that RIF may activate DAF-16 differently and modulate a separate subset of target genes compared to the ones regulated by the IIS pathway.

## Discussion

Drug repositioning is a relatively new area of pharmacology. It deals with discovering new molecular targets of approved drugs so that they may be used to treat morbidities unrelated to the ones for which the drug was developed. Some of the best-known examples of re-engineered drugs include viagra, colesevelam, requip, and gabapentin. Repositioning is profitable as it cuts cost of research and time of development, making drugs available to patients faster and cheaper. In this direction, our study shows that rifampicin and its analog rifamycin SV, developed initially as antibiotics to treat tuberculosis, are effective interventions in extending health and lifespan. Additionally, lifespan extension was also obtained when the drug treatment was started well into adulthood. It will be interesting now to evaluate their efficacy in higher organisms and determine whether RIF treatment late in adulthood can bestow benefits of health as well as longevity on these animals.

Why does RIF and RSV have glycation inhibition properties while RMN does not? A close examination of the chemical structures of RIF, RSV, and RMN revealed that all the three compounds possess macrocyclic lactam and naphthyl moiety fused with a cyclopentanone ring as common features. However, only RIF and RSV possess the *para*-dihydroxy naphthyl moiety (highlighted in red in Fig.1), while RMN has a *para*-aminophenol moiety (highlighted in blue in Fig.1). The *p*-dihydroxyl moiety present in RIF and RSV could undergo spontaneous oxidation to yield highly reactive *p*-quinone structures. On the other hand, the *p*-amino group in RMN cannot form *p*-quinone structures as it is locked up in the fused imidazole ring.

Formation of *p*-quinone structures from the *p*-dihydroxyl moiety could have multiple mechanistic consequences for the observed antiglycation properties of RIF and RSV *in vitro*. First, the quinones, due to their higher reactivity could compete directly with glucose for the primary amino groups on Insulin *in vitro* and other proteins *in vivo*. Benzoquinones and napthaquinones have been shown to inhibit glycation and lipid peroxidation (Jung *et al*., 2005). Secondly, the *p*-quinone moiety could also react with other side chain residues on proteins such as cysteine, tryptophan, and tyrosine *via* a Michael addition reaction, which might decrease the formation of glucosylated Insulin (Schiff's base). Earlier studies using tritium labeling have shown irreversible binding of ^3^H-rifampicin-quinone to albumin which was inhibited by co-incubation with ascorbic acid (Bolt & Remmer, 1976), implicating quinole↔quinone redox system. Additionally, it has been reported that rifampicin quinone, but not reduced rifampicin, showed immunosuppressant activity and prolonged graft acceptance (Konrad & Stenberg, 1988). Therefore, the structure-specific inhibitory activity observed with RIF and RSV could be attributed to the presence of highly reactive quinone moieties. We posit that similar mechanisms may partly explain the robust antiglycating effects of RIF and RSV *in vivo* in *C. elegans*.

We show that the lifespan increase on RIF treatment requires DAF-18/PTEN but not AKT-1 and PDK-1. On the other hand, RIF requires a wild-type *jnk-1* and *jkk-1* allele for its lifespan effects. While the IIS pathway negatively regulates DAF-16, JNK activates it (Oh *et al*., 2005; Wang *et al*., 2005). It is possible that the strong mutant allele of *daf-18* leads to a highly activated IIS pathway and strong DAF-16 sequestration in the cytoplasm. Under this condition, activating JNK (by RIF) is not sufficient to translocate DAF-16 into the nucleus. On the other hand, activating mutations in PDK-1 or AKT-1 may not be sufficiently strong to sequester DAF-16; JNK activation can override this inhibition. It is also possible that DAF-18 and JNK-1 interact independent of PDK-1 or its downstream kinases, by some mechanism as indicated in other systems (Vivanco *et al*., 2007; Hubner *et al*., 2012). As JNK-1 is a stress-associated kinase (Matsukawa *et al*., 2004), RIF treatment may activate a stress response through this cascade, involving its upstream kinase JKK-1, leading to the activation of DAF-16 that results in transcription of genes required for increased longevity. However, this action of RIF alone is not sufficient to increase lifespan as RMN having similar DAF-16 activating property failed to positively affect longevity in wild-type worms. In fact, previous studies have shown that simply activating DAF-16 is not sufficient to increase lifespan (Lin *et al*., 2001). Together, these observations underscores the importance of our finding that a combination of two complementary properties, *viz*. potent glycation inhibition and DAF-16 activation, are required for affecting longevity in a positive manner, as shown by RIF and RSV (Fig.6E).

This dual property of RIF is reflected in its mechanism of action that is both at the transcriptional as well as posttranscriptional level. At the posttranslational level, RIF treatment reduces AGE formation on major cellular proteins that control metabolism and support musculature. At the transcriptional level, it activates genes involved in aging, proteolysis, cell cycle, and cellular detoxification. Interestingly, in both the cases, RIF targets the vitellogenins that are required for growth and development of progeny. However, the vitellogenin proteins appear to be toxic at old age in worms as they undergo AGE modifications, and knockdown of *vit-2* as well as *vit-5* by RNAi increases lifespan (Murphy *et al*., 2003). Vitellogenins are required during the self-fertile reproductive phase of hermaphrodites and later for reproduction by cross-fertilization. In the absence of cross-fertilization in older worms as well as in aging *Drosophila*, vitellogenins accumulate in the body cavity and are subjected to oxidative damage. In later life, they become one of the most prevalent proteins in aggregates (Nakamura *et al*., 1999; David *et al*., 2010; McGee *et al*., 2011). AGE modifications on these proteins may accelerate aggregation and toxicity. Thus, decreased vitellogenin protein accumulation and reduced AGE modification may contribute to extended longevity imparted by RIF. Additionally, we found that several genes involved in xenobiotic detoxification are upregulated on RIF treatment. These genes have recently been implicated in lifespan regulation (Shore & Ruvkun, 2013) and may constitute one of the arms through which RIF has a positive effect on longevity. From the proteomics data, we found that proteins important in muscle function have several AGE modifications that are reduced on RIF treatment. These proteins include myosin, tropomyosin, paramyosin as well as actin and tubulin. By reducing glycation on major muscle-specific proteins, RIF may positively affect muscle function as evidenced in our paralysis assays. Apart from these proteins, enzymes involved in important cellular processes including glycolysis (Glyceraldehyde 3-phosphate dehydrogenase), Kreb's cycle (citrate synthase, aconitase), and amino acid metabolism (arginine kinase, methylmalonate semialdehyde dehydrogenase) develop lower number of AGE modifications in the presence of RIF. This may lead to efficient metabolism required for extended lifespan as observed on RIF treatment. Thus, the multidimensional effects of RIF result in one of the most dramatic effects of a drug on longevity in *C. elegans*.

In conclusion, our study using the most suitable model organism for aging research shows how structure-dependent differential activity of three FDA-approved drugs may help in repositioning them to potentially treat age-associated pathologies and increase longevity.

## Experimental procedures

Detailed experimental procedures are provided as Data S1. Worms were bleached, and eggs were placed on OP50-Amp seeded NGM-Amp plates. They were grown at 20 °C till young adult stage, rinsed off, and washed 4 times with M9 buffer. Thereafter, worms were resuspended in S-complete buffer and diluted further to contain 10–15 worms per 10 μL. For each experiment, six-eight wells were used for the same treatment and controls were set up in each plate. Thus, the total number of worms was ∼60 at the beginning of the experiments. Bacteria for liquid culture were prepared using 14 h/37 °C-grown OP50-Amp that was resuspended in S-complete to 100 mg mL^−1^ and heat-killed at 100 °C for 45 min. A master mix was then prepared such that each well of a noncoated sterile 96-well culture plate contained 80 μL S-complete, 10 μL resuspended worms, 9.9 μL heat-killed OP50-Amp, 1.6 μL carbenicillin (50 mg mL^−1^ stock), and 0.6 μL amphotericin B (250 μg mL^−1^ stock). Later, to each well, 30 μL of 0.6 mm 5-Fluoro-2′-deoxyuridine (FuDr; Sigma-Aldrich, St. Louis, MO, USA) was added and, the next day, 10 μL of a solution in S-complete containing the drugs (dissolved in DMSO, stored at 4 °C) or only DMSO was added. The worms were fed again on the 5th day with 9 μL heat-killed OP50-Amp/well. Every other day, the worms in the wells were exposed to light from the diascopic base of a stereozoom microscope and the plates gently agitated by tapping; based on their movement, the worms were scored as dead or alive. Dead worms were not extracted from the wells; a count of total number of live worms in each well was kept to plot the lifespan. Lifespans were repeated multiple times, and consolidated data are shown (details in Table s3). *In vivo* AGE modification of proteins was determined by LC-MS^E^, a data-independent acquisition. For proteotoxicity assay, CL2006 (Link, 1995) was grown as above and worms from a single well were transferred to an OP50-seeded NGM plate for scoring. For protein extraction, in a 250-mL flask, 17.78 mL S-complete, 2.20 mL worm suspension (∼5000 worms), 1.9 mL heat-killed OP50-Amp, 33 μL Carbenicillin, 13.2 μL amphotericin B, and 6.6 mL of 0.6 mM FuDr was added. The next day, 2.2 mL of S-complete containing 200 μm RIF (dissolved in DMSO) or only 0.2% DMSO with or without glucose was added and grown for the indicated number of days at 20 °C before harvesting. For qRT–PCR analysis, cDNA was synthesized using 5 μg of total RNA and SuperScript III cDNA synthesis kit (Life Technologies, Carlsbad, CA, USA). Microarray analysis was carried using an Affymetrix, USA platform. Data were analyzed using Genespring GX 11.1 with Affymetrix default analysis settings. The microarray data are available at http://www.ncbi.nlm.nih.gov/geo/query/acc.cgi?token=xjcdjagswaygerq&acc=GSE45292. Statistical analyses for survival were conducted using Mantel–Cox log-rank test with oasis software available at http://sbi.postech.ac.kr/oasis. *P* value calculations for other experiments were performed by unpaired *t*-test using sigmaplot 10.0 (Systat Software, Inc., San Jose, CA, USA).

## Supporting Information

**Data S1** Supplementary Materials and methods **Fig. S1** (A) Pathways by which AGEs are formed in animal cells, as collated from Singh *et al*., 2001; Ulrich & Cerami, 2005 and Ahmed, 2001. **Fig. S2** (A) RIF increased life span on live *E. coli* OP50 in a concentration dependent manner. **Fig. S3** (A) The life span of *glod-4* mutant allele, *glod-4(gk189)* is significantly shorter than WT. **Fig. S4** RIF increased life span of activated PDK-1 strain, *pdk-1(mg142)*. **Fig. S5***(A)* GO term analysis of genes that were significantly downregulated upon RIF treatment. **Table s1** List of genes upregulated on Rifampicin treatment determined by Microarray analysis along with the number of DAF-16 binding sites in their 5 kb upstream region. **Table S2** List of genes downregulated on Rifampicin treatment determined by Microarray analysis along with the number of DAF-16 binding sites in their 5 kb upstream region. **Table S3** Details of life spans that are reported in the manuscript. **Table S4** Relative quantification of fold changes of proteins detected by LC-MS^E^.

## References

[b1] Ahmed N (2005). Advanced glycation endproducts–role in pathology of diabetic complications. Diabetes Res. Clin. Pract.

[b2] Blackburn K, Goshe MB (2009). Challenges and strategies for targeted phosphorylation site identification and quantification using mass spectrometry analysis. Brief. Funct. Genomic Proteomic.

[b3] Bolt HM, Remmer H (1976). Implication of rifampicin-quinone in the irreversible binding of rifampicin to macromolecules. Xenobiotica.

[b4] Brummer G, Littlechild S, McCall S, Zhang Y, Conrad GW (2011). The role of nonenzymatic glycation and carbonyls in collagen cross-linking for the treatment of keratoconus. Invest. Ophthalmol. Vis. Sci.

[b5] David DC, Ollikainen N, Trinidad JC, Cary MP, Burlingame AL, Kenyon C (2010). Widespread protein aggregation as an inherent part of aging in C. elegans. PLoS Biol.

[b6] Dillin A, Crawford DK, Kenyon C (2002). Timing requirements for insulin/IGF-1 signaling in *C. elegans*. Science.

[b7] Golegaonkar SB, Bhonsle HS, Boppana R, Kulkarni MJ (2010). Discovery of rifampicin as a new anti-glycating compound by matrix-assisted laser desorption/ionization mass spectrometry-based insulin glycation assay. Eur. J. Mass Spectrom. (Chichester, Eng).

[b8] Henderson ST, Johnson TE (2001). daf-16 integrates developmental and environmental inputs to mediate aging in the nematode *Caenorhabditis elegans*. Curr. Biol.

[b9] Hipkiss AR (2006). Accumulation of altered proteins and ageing: causes and effects. Exp. Gerontol.

[b10] Hubner A, Mulholland DJ, Standen CL, Karasarides M, Cavanagh-Kyros J, Barrett T, Chi H, Greiner DL, Tournier C, Sawyers CL, Flavell RA, Wu H, Davis RJ (2012). JNK and PTEN cooperatively control the development of invasive adenocarcinoma of the prostate. Proc. Natl. Acad. Sci. USA.

[b11] Jung YS, Joe BY, Cho SJ, Konishi Y (2005). 2,3-Dimethoxy-5-methyl-1,4-benzoquinones and 2-methyl-1,4-naphthoquinones: glycation inhibitors with lipid peroxidation activity. Bioorg. Med. Chem. Lett.

[b12] Kenyon CJ (2010). The genetics of ageing. Nature.

[b13] enyon C, Murphy CT (2006). Enrichment of regulatory motifs upstream of predicted DAF-16 targets. Nat. Genet.

[b14] Khalifah RG, Baynes JW, Hudson BG (1999). Amadorins: novel post-Amadori inhibitors of advanced glycation reactions. Biochem. Biophys. Res. Commun.

[b15] Konrad P, Stenberg P (1988). Rifampicin quinone is an immunosuppressant, but not rifampicin itself. Clin. Immunol. Immunopathol.

[b16] Lin K, Dorman JB, Rodan A, Kenyon C (1997). *daf-16*: an HNF-3/forkhead family member that can function to double the life-span of *Caenorhabditis elegans*. Science.

[b17] Lin K, Hsin H, Libina N, Kenyon C (2001). Regulation of the Caenorhabditis elegans longevity protein DAF-16 by insulin/IGF-1 and germline signaling. Nat. Genet.

[b18] Link CD (1995). Expression of human beta-amyloid peptide in transgenic *Caenorhabditis elegans*. Proc. Natl. Acad. Sci. USA.

[b19] Luevano-Contreras C, Chapman-Novakofski K (2010). Dietary advanced glycation end products and aging. Nutrients.

[b20] Matsukawa J, Matsuzawa A, Takeda K, Ichijo H (2004). The ASK1-MAP kinase cascades in mammalian stress response. J. Biochem.

[b21] McGee MD, Weber D, Day N, Vitelli C, Crippen D, Herndon LA, Hall DH, Melov S (2011). Loss of intestinal nuclei and intestinal integrity in aging *C. elegans*. Aging Cell.

[b22] Mihaylova VT, Borland CZ, Manjarrez L, Stern MJ, Sun H (1999). The PTEN tumor suppressor homolog in *Caenorhabditis elegans* regulates longevity and dauer formation in an insulin receptor-like signaling pathway. Proc. Natl. Acad. Sci. USA.

[b23] Morcos M, Du X, Pfisterer F, Hutter H, Sayed AA, Thornalley P, Ahmed N, Baynes J, Thorpe S, Kukudov G, Schlotterer A, Bozorgmehr F, El Baki RA, Stern D, Moehrlen F, Ibrahim Y, Oikonomou D, Hamann A, Becker C, Zeier M, Schwenger V, Miftari N, Humpert P, Hammes HP, Buechler M, Bierhaus A, Brownlee M, Nawroth PP (2008). Glyoxalase-1 prevents mitochondrial protein modification and enhances lifespan in *Caenorhabditis elegans*. Aging Cell.

[b24] Murphy CT, McCarroll SA, Bargmann CI, Fraser A, Kamath RS, Ahringer J, Li H, Kenyon C (2003). Genes that act downstream of DAF-16 to influence the lifespan of *Caenorhabditis elegans*. Nature.

[b25] Nakamura A, Yasuda K, Adachi H, Sakurai Y, Ishii N, Goto S (1999). Vitellogenin-6 is a major carbonylated protein in aged nematode, *Caenorhabditis elegans*. Biochem. Biophys. Res. Commun.

[b26] Nedic O, Rattan SI, Grune T, Trougakos IP (2013). Molecular effects of advanced glycation end products on cell signalling pathways, ageing and pathophysiology. Free Radical Res.

[b27] Ogg S, Ruvkun G (1998). The C. elegans PTEN homolog, DAF-18, acts in the insulin receptor-like metabolic signaling pathway. Mol. Cell.

[b28] Ogg S, Paradis S, Gottlieb S, Patterson GI, Lee L, Tissenbaum HA, Ruvkun G (1997). The Fork head transcription factor DAF-16 transduces insulin-like metabolic and longevity signals in *C. elegans*. Nature.

[b29] Oh SW, Mukhopadhyay A, Svrzikapa N, Jiang F, Davis RJ, Tissenbaum HA (2005). JNK regulates lifespan in *Caenorhabditis elegans* by modulating nuclear translocation of forkhead transcription factor/DAF-16. Proc. Natl. Acad. Sci. USA.

[b30] Oh SW, Mukhopadhyay A, Dixit BL, Raha T, Green MR, Tissenbaum HA (2006). Identification of direct DAF-16 targets controlling longevity, metabolism and diapause by chromatin immunoprecipitation. Nat. Genet.

[b31] Paradis S, Ruvkun G (1998). Caenorhabditis elegans Akt/PKB transduces insulin receptor-like signals from AGE-1 PI3 kinase to the DAF-16 transcription factor. Genes Dev.

[b32] Paradis S, Ailion M, Toker A, Thomas JH, Ruvkun G (1999). A PDK1 homolog is necessary and sufficient to transduce AGE-1 PI3 kinase signals that regulate diapause in *Caenorhabditis elegans*. Genes Dev.

[b33] Rabbani N, Thornalley PJ (2011). Glyoxalase in diabetes, obesity and related disorders. Semin. Cell Dev. Biol.

[b34] Semba RD, Nicklett EJ, Ferrucci L (2010). Does accumulation of advanced glycation end products contribute to the aging phenotype?. J. Gerontol. A Biol. Sci. Med. Sci.

[b35] Shore DE, Ruvkun G (2013). A cytoprotective perspective on longevity regulation. Trends Cell Biol.

[b36] Silva JC, Denny R, Dorschel C, Gorenstein MV, Li GZ, Richardson K, Wall D, Geromanos SJ (2006). Simultaneous qualitative and quantitative analysis of the *Escherichia coli* proteome: a sweet tale. Mol. Cell Proteomics.

[b37] Singh R, Barden A, Mori T, Beilin L (2001). Advanced glycation end-products: a review. Diabetologia.

[b38] Sodhi CP, Rana S, Mehta S, Vaiphei K, Goel RC, Mehta SK (1997). Study of oxidative-stress in rifampicin-induced hepatic injury in growing rats with and without protein-energy malnutrition. Hum. Exp. Toxicol.

[b39] Thornalley PJ (2008). Protein and nucleotide damage by glyoxal and methylglyoxal in physiological systems–role in ageing and disease. Drug Metabol. Drug Interact.

[b40] Ulrich P, Cerami A (2001). Protein glycation, diabetes, and aging. Recent Prog. Horm. Res.

[b41] Van Nostrand EL, Sanchez-Blanco A, Wu B, Nguyen A, Kim SK (2013). Roles of the developmental regulator unc-62/homothorax in limiting longevity in *Caenorhabditis elegans*. PLoS Genet.

[b42] Vivanco I, Palaskas N, Tran C, Finn SP, Getz G, Kennedy NJ, Jiao J, Rose J, Xie W, Loda M, Golub T, Mellinghoff IK, Davis RJ, Wu H, Sawyers CL (2007). Identification of the JNK signaling pathway as a functional target of the tumor suppressor PTEN. Cancer Cell.

[b43] Wang MC, Bohmann D, Jasper H (2005). JNK extends life span and limits growth by antagonizing cellular and organism-wide responses to insulin signaling. Cell.

[b44] Xie H, Gilar M, Gebler JC (2009). Characterization of protein impurities and site-specific modifications using peptide mapping with liquid chromatography and data independent acquisition mass spectrometry. Anal. Chem.

[b45] Xue M, Rabbani N, Thornalley PJ (2011). Glyoxalase in ageing. Semin. Cell Dev. Biol.

