# Supplementary material for: Rifampicin reduces advanced glycation end products and activates DAF-16 to increase lifespan in Caenorhabditis elegans
Source: Aging Cell. 2015 Feb 26;14(3):463–73. doi: 10.1111/acel.12327 (PMC4406675; doi:10.1111/acel.12327)
Supplement: Supplementary file 1 [file acel0014-0463-sd1.zip › Golegaonkar_Figures Aging Cell_010115_SUP.pptx]

## Slide 1
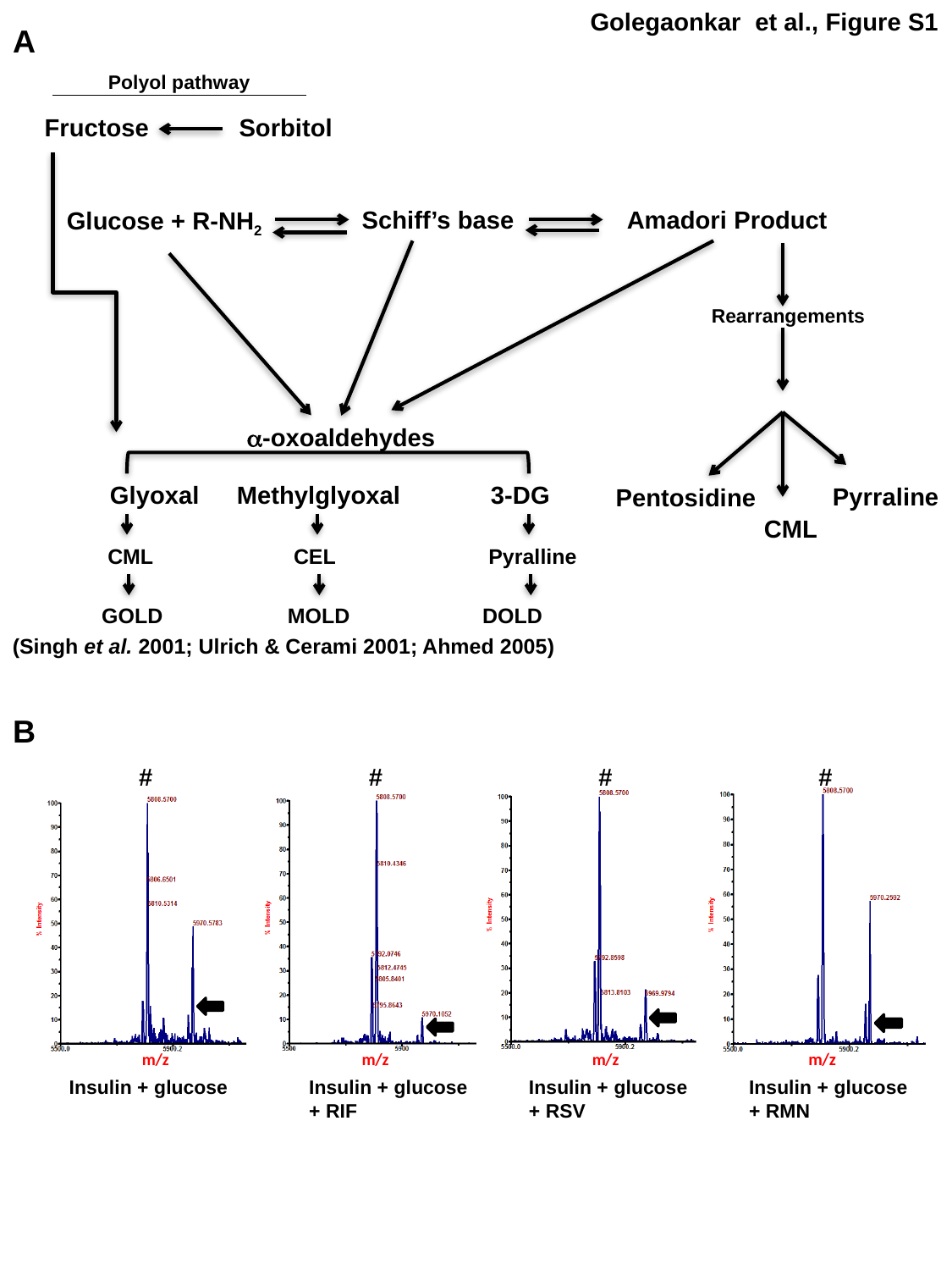

Golegaonkar et al., Figure S1
A
Polyol pathway
Fructose
Sorbitol
Glucose + R-NH2
Schiff’s base
Amadori Product
Rearrangements
a-oxoaldehydes
Glyoxal	Methylglyoxal	3-DG
Pyrraline
Pentosidine
CML
CML	 CEL		Pyralline
GOLD	 MOLD		DOLD
(Singh et al. 2001; Ulrich & Cerami 2001; Ahmed 2005)
B
#
#
#
#
Insulin + glucose
Insulin + glucose
+ RIF
Insulin + glucose
+ RSV
Insulin + glucose
+ RMN

## Slide 2
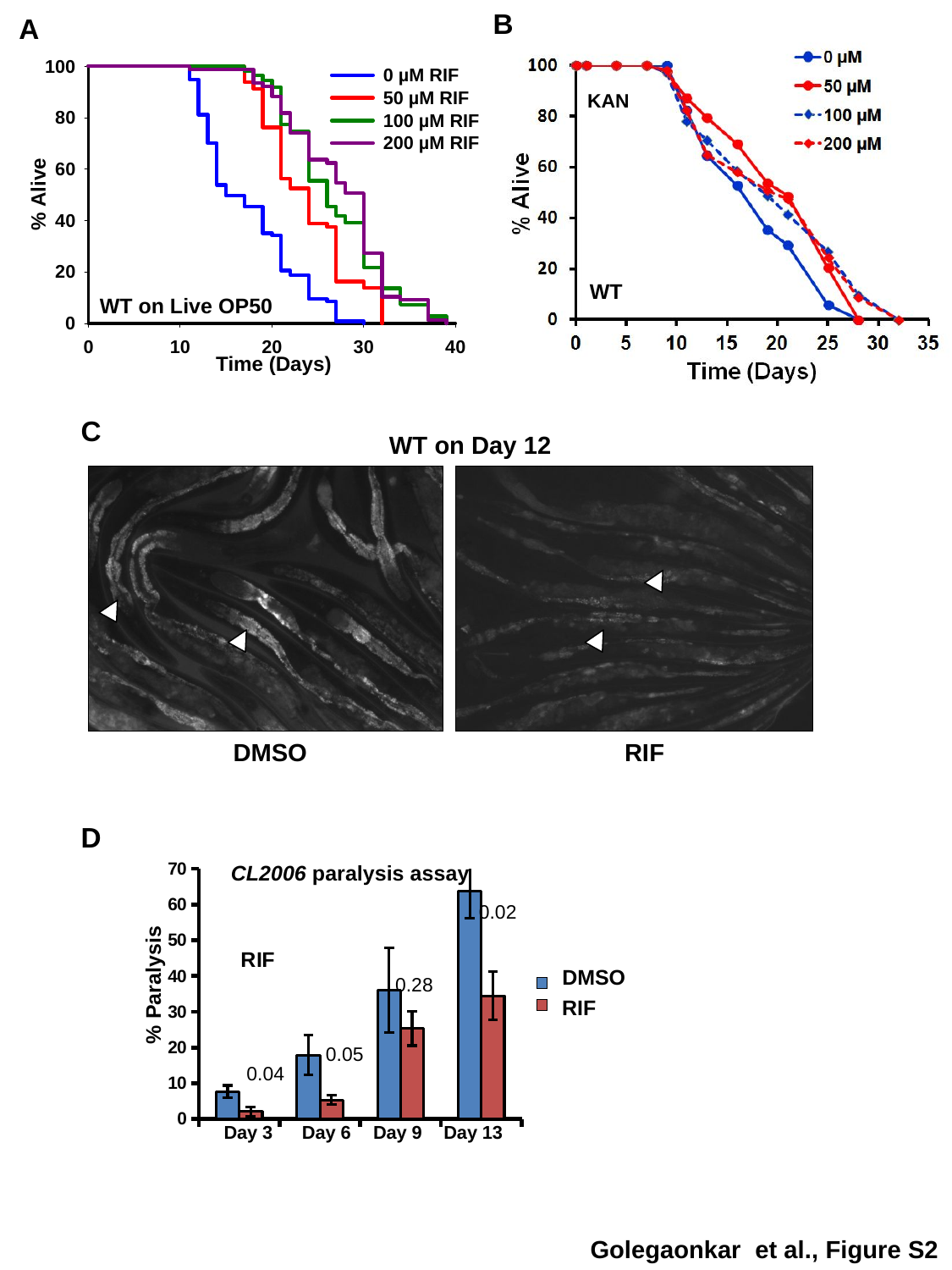

B
A
KAN
WT
WT on Live OP50
C
WT on Day 12
DMSO
RIF
D
### Chart
| Category | | |
|---|---|---|CL2006 paralysis assay
0.02
DMSO
% Paralysis
0.28
RIF
0.05
0.04
Day 3
Day 6
Day 9
Day 13
Golegaonkar et al., Figure S2

## Slide 3
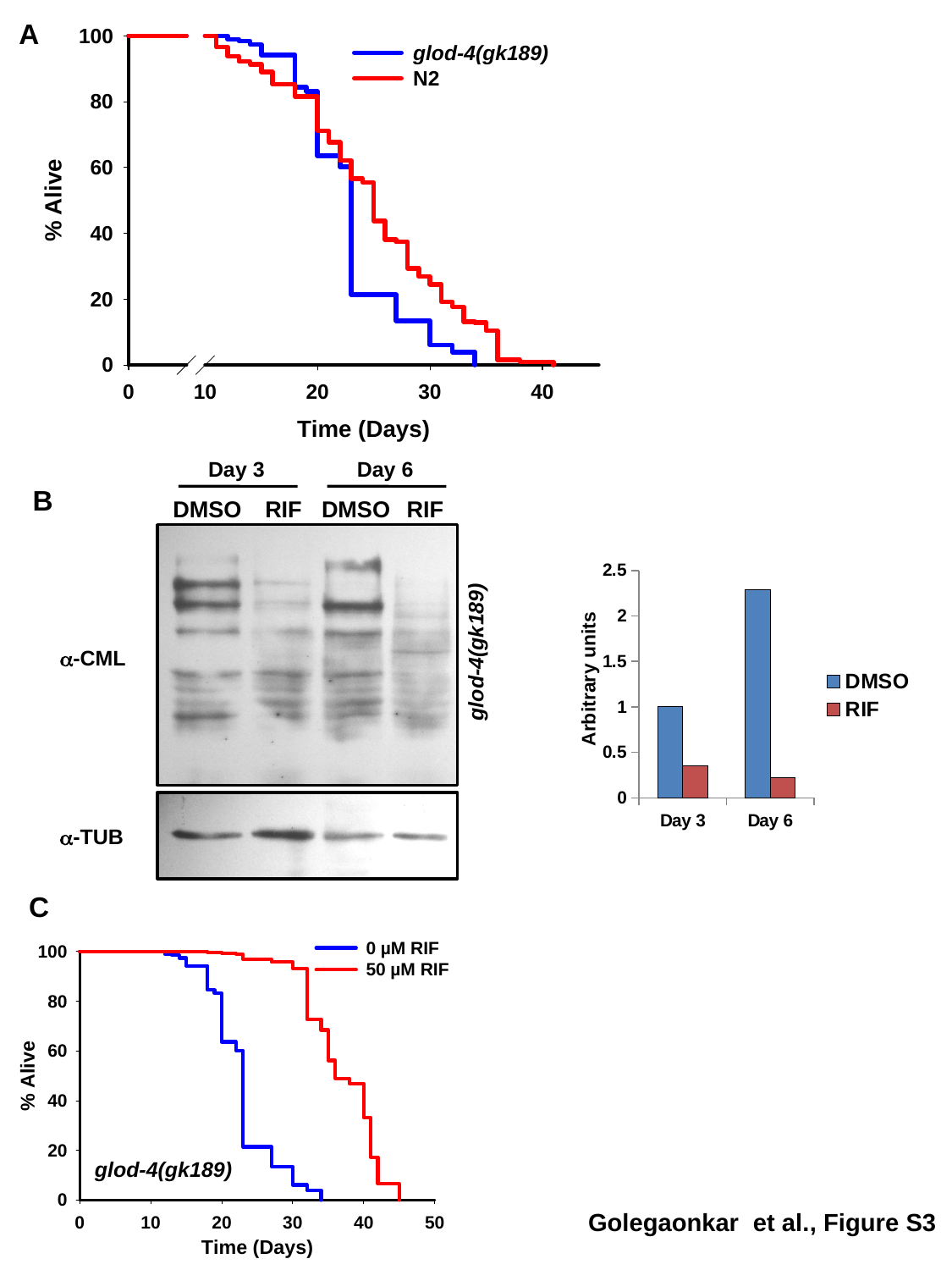

A
Day 3
Day 6
B
DMSO
RIF
DMSO
RIF
### Chart
| Category | DMSO | RIF |
|---|---|---|
| Day 3 | 1.0 | 0.353677653797063 |
| Day 6 | 2.285439713185339 | 0.22498325149099843 |glod-4(gk189)
a-CML
Arbitrary units
a-TUB
C
glod-4(gk189)
Golegaonkar et al., Figure S3

## Slide 4
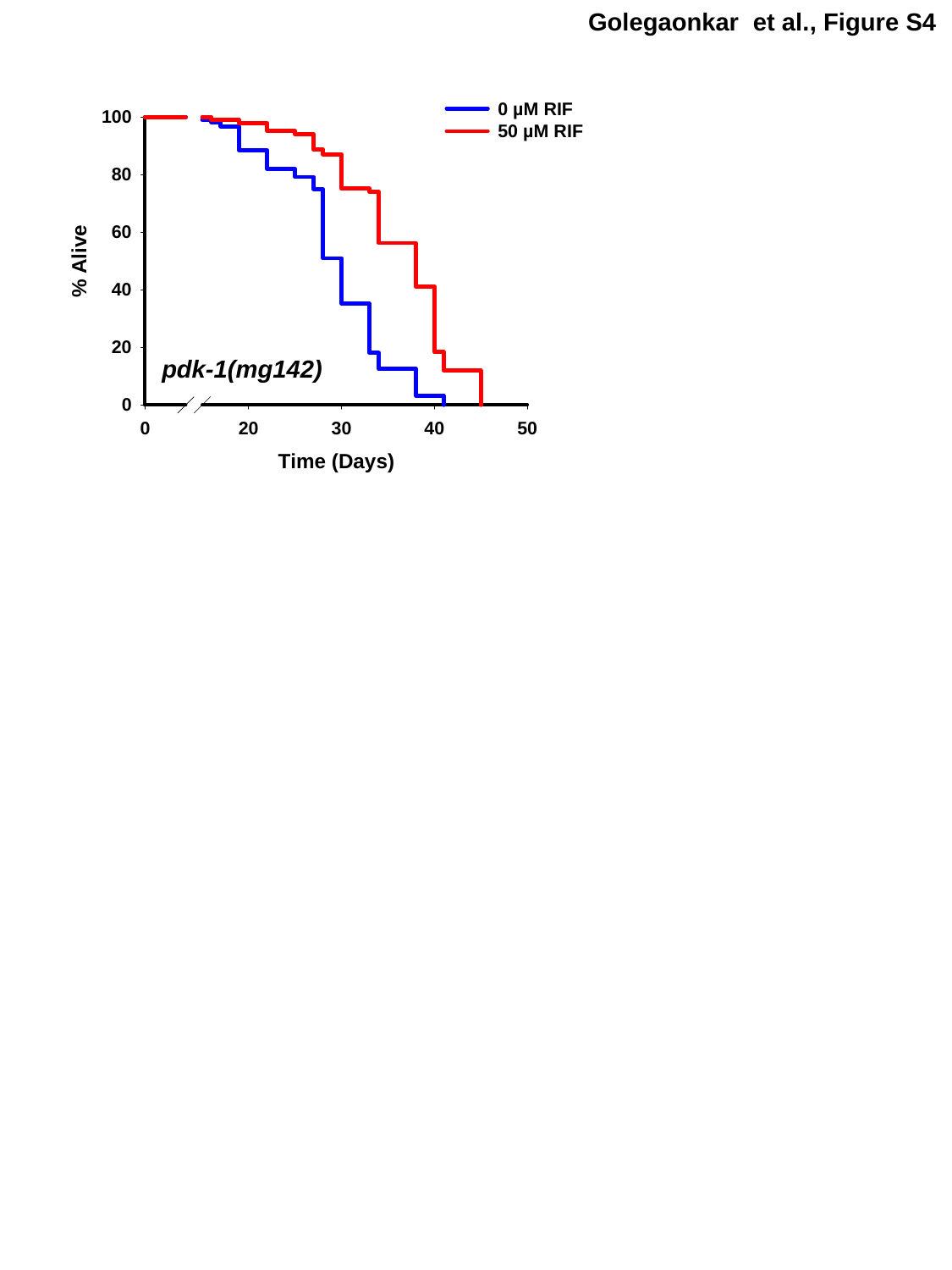

Golegaonkar et al., Figure S4
pdk-1(mg142)

## Slide 5
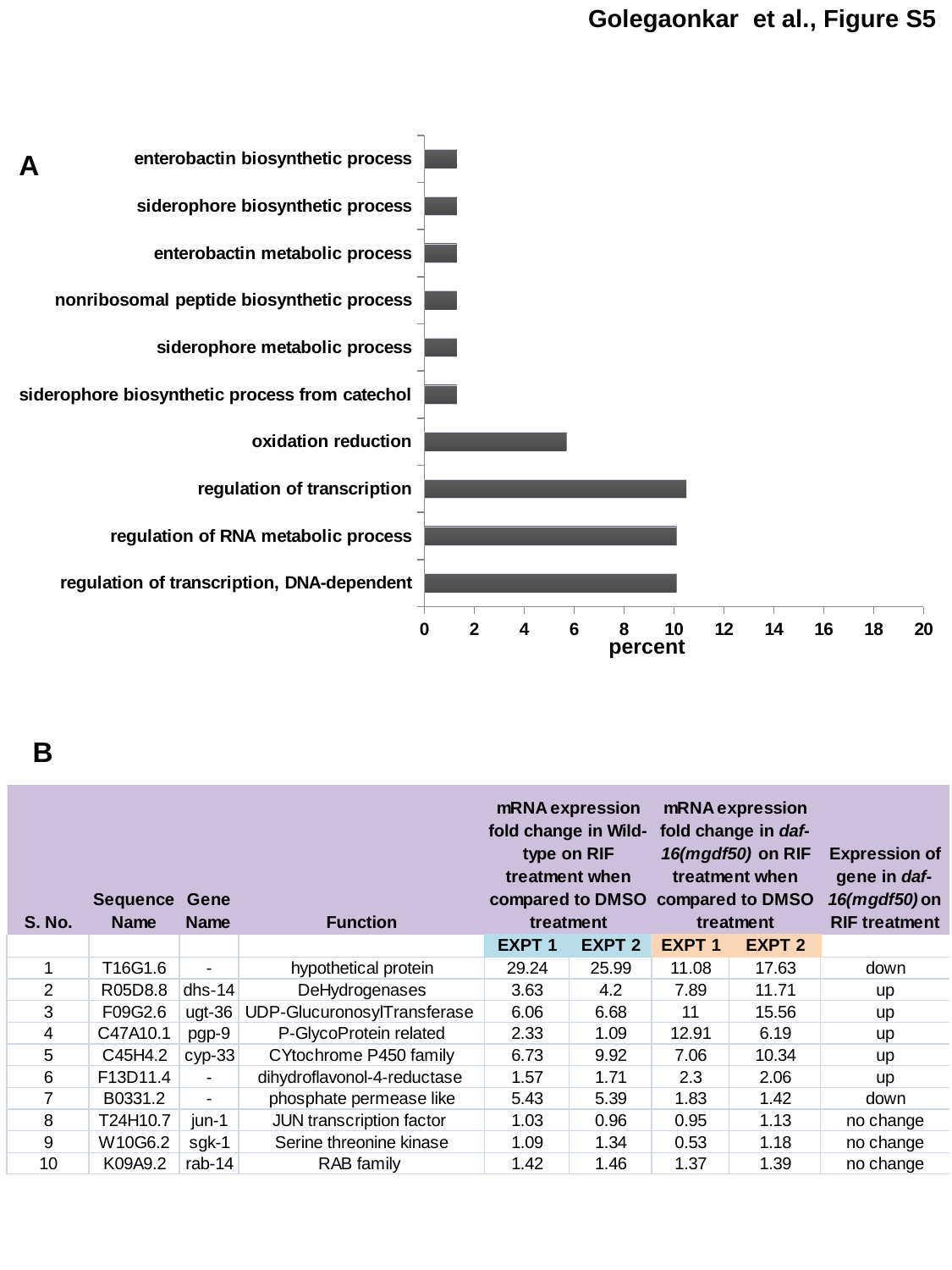

Golegaonkar et al., Figure S5
### Chart
| Category | |
|---|---|
| regulation of transcription, DNA-dependent | 10.1 |
| regulation of RNA metabolic process | 10.1 |
| regulation of transcription | 10.5 |
| oxidation reduction | 5.7 |
| siderophore biosynthetic process from catechol | 1.3 |
| siderophore metabolic process | 1.3 |
| nonribosomal peptide biosynthetic process | 1.3 |
| enterobactin metabolic process | 1.3 |
| siderophore biosynthetic process | 1.3 |
| enterobactin biosynthetic process | 1.3 |A
percent
B
