# Supplementary material for: Rifampicin reduces advanced glycation end products and activates DAF-16 to increase lifespan in Caenorhabditis elegans
Source: Aging Cell. 2015 Feb 26;14(3):463–73. doi: 10.1111/acel.12327 (PMC4406675; doi:10.1111/acel.12327)
Supplement: Supplementary file 1 [file acel0014-0463-sd1.zip › Golegaonkar_Table S3_07012015.doc]

| **Table S3: Details of life spans that are reported in the manuscript** | | | | | | |
| --- | --- | --- | --- | --- | --- | --- |
| **Genetic background** | **Treatment** | **Mean ± SEM** | **No. of Subjects** | **Percent increase (+) or decrease (-) w.r.t. control** | ***P*-value** | **Number of expt** |
| ***Wild Type*** | 0 µM RIF | 17.47 ± 0.47 | 117 |  |  | 2 |
|  | 50 µM RIF | 23.90 ± 0.52 | 80 | (+) 36.806 | ≤0.0001 | 2 |
|  | 100 µM RIF | 26.82 ± 0.51 | 110 | (+) 53.520 | ≤0.0001 | 2 |
|  | 200 µM RIF | 27.45 ± 0.64 | 77 | (+) 57.127 | ≤0.0001 | 2 |
|  |  |  |  |  |  |  |
| ***Wild Type*** | 0 µM RIF | 24.89 ± 0.28 | 644 |  |  | 10 |
|  | 50 µM RIF | 39.57 ± 0.29 | 625 | (+) 58.980 | ≤0.0001 | 10 |
|  | 50 µM RSV | 30.09 ± 0.61 | 159 | (+) 20.892 | ≤0.0001 | 3 |
|  | 50 µM RMN | 21.48 ± 0.46 | 320 | (-) 13.700 | ≤0.0001 | 3 |
|  |  |  |  |  |  |  |
|  | 0 µM RIF 5 Day | 28.20 ± 0.51 | 136 |  |  | 3 |
|  | 50 µM RIF 5 Day | 41.01 ± 0.93 | 121 | (+) 45.426 | ≤0.0001 | 3 |
|  |  |  |  |  |  |  |
|  | 0 µM RIF 9 Day | 28.62 ± 0.50 | 138 |  |  | 3 |
|  | 50 µM RIF 9 Day | 39.46 ± 0.88 | 140 | (+) 37.876 | ≤0.0001 | 3 |
|  |  |  |  |  |  |  |
| ***daf-16*** | 0 µM RIF | 17.80 ± 0.25 | 191 |  |  | 3 |
|  | 50 µM RIF | 17.11 ± 0.25 | 163 | (-) 3.876 | 0.122 | 3 |
|  |  |  |  |  |  |  |
| ***daf-18*** | 0 µM RIF | 18.60 ± 0.26 | 276 |  |  | 3 |
|  | 50 µM RIF | 19.84 ± 0.21 | 305 | (+) 6.667 | 0.0739 | 3 |
|  |  |  |  |  |  |  |
| ***akt-1*** | 0 µM RIF | 18.55 ± 0.22 | 178 |  |  | 2 |
|  | 50 µM RIF | 36.96 ± 0.70 | 108 | (+) 99.245 | ≤0.0001 | 2 |
|  |  |  |  |  |  |  |
| ***jnk-1*** | 0 µM RIF | 39.52 ± 0.61 | 242 |  |  | 4 |
|  | 50 µM RIF | 39.14 ± 0.51 | 287 | (+) 0.698 | 0.6307 | 4 |
|  |  |  |  |  |  |  |
| ***jkk-1*** | 0 µM RIF | 33.38 ± 0.43 | 182 |  |  | 3 |
|  | 50 µM RIF | 33.96 ± 0.47 | 161 | (+) 1.738 | 0.3088 | 3 |
|  |  |  |  |  |  |  |
| ***glod-4*** | 0 µM RIF | 22.78 ± 0.23 | 379 |  |  | 3 |
|  | 50 µM RIF | 36.88 ± 0.31 | 278 | (+) 61.896 | ≤0.0001 | 3 |
|  |  |  |  |  |  |  |
| ***pdk-1*** | 0 µM RIF | 29.18 ± 0.40 | 216 |  |  | 3 |
|  | 50 µM RIF | 35.99 ± 0.47 | 185 | (+) 23.338 | ≤0.0001 | 3 |
|  |  |  |  |  |  |  |
|  |  |  |  |  |  |  |
| **Wild Type** | N2 RIF 0 | 28.17 ± 0.58 | 223 |  |  | 3 |
|  | N2 RIF 1 | 37.96 ± 0.50 | 183 | (+) 34.753 | ≤0.0001 | 3 |
|  | N2 RIF  5 | 40.18 ± 0.55 | 165 | (+) 42.634 | ≤0.0001 | 3 |
|  | N2 RIF 10 | 40.94 ± 0.61 | 173 | (+) 45.332 | ≤0.0001 | 3 |
|  | N2 RIF 20 | 42.48 ± 0.60 | 187 | (+) 50.799 | ≤0.0001 | 3 |
|  | N2 RIF 30 | 43.98 ± 0.56 | 177 | (+) 56.124 | ≤0.0001 | 3 |
|  | N2 RIF 50 | 43.38 ± 0.52 | 254 | (+) 53.994 | ≤0.0001 | 3 |
|  | N2 RIF 100 | 41.38 ± 0.53 | 201 | (+) 46.894 | ≤0.0001 | 3 |
|  | N2 RIF 200 | 38.88 ± 0.48 | 197 | (+) 38.019 | ≤0.0001 | 3 |
|  |  |  |  |  |  |  |
