# Supplementary material for: Rifampicin reduces advanced glycation end products and activates DAF-16 to increase lifespan in Caenorhabditis elegans
Source: Aging Cell. 2015 Feb 26;14(3):463–73. doi: 10.1111/acel.12327 (PMC4406675; doi:10.1111/acel.12327)
Supplement: Supplementary file 1 [file acel0014-0463-sd1.zip › Golegaonkar_Table S2_07012015.doc]

|  |  |  | **Fold**  **change** | **DAF-16 binding site motif** | |
| --- | --- | --- | --- | --- | --- |
| **Sequence Name** | **Gene Name** | **Annotated function** |  | **Canonical** | **DAE** |
|  |  |  |  | **GTAAAC/TA** | **CTTATCA** |
| C57430 | *ilys-5* | Invertebrate LYSozyme | -53.71 | 3 | 1 |
| C17H12.8 |  | hypothetical protein | -49.38 | 0 | 3 |
| C32H11.12 | *dod-24* | Downstream Of DAF-16 | -47.47 | 1 | 2 |
| F55G11.5 | *dod-22* | Downstream Of DAF-16 | -12.31 | 3 | 4 |
| C14C6.5 |  | hypothetical protein | -12.05 | 0 | 0 |
| F55G11.4 |  | hypothetical protein | -10.47 | 3 | 3 |
| F15E11.14 |  | hypothetical protein | -10.22 | 1 | 1 |
| C12C8.1 | *hsp-70* | Heat Shock Protein | -9.56 | 2 | 0 |
| F56D6.2 | *clec-67* | C-type LECtin | -8.85 | 1 | 2 |
| Y105C5B.15 |  | hypothetical protein | -8.69 | 3 | 2 |
| F01D5.5 |  | hypothetical protein | -7.89 | 2 | 2 |
| F35E8.13 |  | hypothetical protein | -7.64 | 3 | 1 |
| F49F1.1 |  | hypothetical protein | -7.39 | 1 | 2 |
| F01D5.1 |  | hypothetical protein | -6.82 | 1 | 5 |
| C45B2.1 |  | hypothetical protein | -6.76 | 1 | 1 |
| AU113368 | *clec-85* | C-type LECtin | -6.46 | 1 | 0 |
| C32H11.4 |  | hypothetical protein | -6.41 | 5 | 2 |
| F42A10.6 |  | hypothetical protein | -6.29 | 3 | 4 |
| F53E10.4 | *irg-3* | hypothetical protein | -6.18 | 0 | 1 |
| ZK896.7 | *clec-186* | C-type LECtin | -6.09 | 1 | 2 |
| C02A12.4 | *lys-7* | LYSozyme | -5.81 | 2 | 1 |
| AU112775 |  | hypothetical protein | -5.78 | 3 | 1 |
| F55G11.8 |  | hypothetical protein | -5.64 | 1 | 3 |
| F40G12.5 |  | hypothetical protein | -5.25 | 3 | 0 |
| C05C10.4 | *pho-11* | hypothetical protein | -5.11 | 1 | 1 |
| F57F4.4 |  | hypothetical protein | -5.09 | 1 | 3 |
| Y49E10.18 |  | hypothetical protein | -5.08 | 0 | 3 |
| C50B6.7 |  | amylase | -5.07 | 1 | 1 |
| F01D5.2 |  | hypothetical protein | -5.04 | 2 | 4 |
| C03E10.6 | *clec-222* | C-type LECtin | -4.65 | 2 | 2 |
| ZK1320.2 |  | hypothetical protein | -4.55 | 4 | 2 |
| Y19D10B.A |  | hypothetical protein | -4.43 | 2 | 0 |
| K10C2.3 |  | aspartyl protease | -4.42 | 1 | 0 |
| Y53F4B.30 | *gst-27* | Glutathione S-Transferase | -4.40 | 1 | 2 |
| T08A9.7 | *spp-3* | SaPosin-like Protein family | -4.22 | 1 | 5 |
| B0365.6 | *clec-41* | C-type LECtin | -4.19 | 1 | 0 |
| Y102A11A.D |  | hypothetical protein | -3.97 | 4 | 0 |
| W10G11.2 |  | hypothetical protein | -3.78 | 2 | 1 |
| F28H7.3 |  | lipase | -3.73 | 1 | 3 |
| Y34B4A.C |  | hypothetical protein | -3.72 | 0 | 4 |
| C51901 | *unc-15* | UNCoordinated | -3.57 | 1 | 1 |
| ZK1320.3 |  | hypothetical protein | -3.53 | 3 | 1 |
| W10G11.3 |  | hypothetical protein | -3.30 | 1 | 1 |
| CEC7564 | *vit-4* | VITellogenin structural gene | -3.27 | 1 | 3 |
| F59D8.A | *vit-3* | VITellogenin structural gene | -3.24 | 1 | 1 |
| C04F6.1 | *vit-5* | VITellogenin structural gene | -3.22 | 1 | 1 |
| F35C5.8 | *clec-65* | C-type LECtin | -3.19 | 0 | 2 |
| Y71G12A.A |  | hypothetical protein | -3.19 | 2 | 2 |
| C58535 |  | hypothetical protein | -3.18 | 4 | 1 |
| W04E12.8 | *clec-50* | C-type LECtin | -3.11 | 1 | 1 |
| F57F5.1 |  | cysteine protease | -3.11 | 2 | 1 |
| CEC3896 | *nspc-2* | Nematode Specific Peptide family | -3.08 | 1 | 0 |
| C17G10.5 | *lys-8* | LYSozyme | -3.07 | 4 | 0 |
| F56A4.2 |  | C-type LECtin | -3.03 | 2 | 1 |
| F56F10.1 |  | hypothetical protein | -2.99 | 2 | 0 |
| F55F3.2 |  | hypothetical protein | -2.89 | 3 | 3 |
| ZK1320.1 | *gstk-1* | Glutathione S-Transferase Kappa protein | -2.78 | 0 | 2 |
| ZK896.5 |  | hypothetical protein | -2.76 | 2 | 1 |
| F52E1.5 |  | hypothetical protein | -2.70 | 2 | 2 |
| F49C12.14 |  | hypothetical protein | -2.67 | 0 | 2 |
| F27E5.1 |  | hypothetical protein | -2.60 | 2 | 0 |
| C30F12.7 |  | hypothetical protein | -2.59 | 1 | 2 |
| K02F3.9 |  | hypothetical protein | -2.52 | 4 | 0 |
| M28.4 |  | hypothetical protein | -2.49 | 0 | 3 |
| R09A8.4 | *col-182* | COLlagen | -2.47 | 1 | 1 |
| Y38F1A.9 | *oig-2* | One IG domain | -2.46 | 0 | 0 |
| Y39B6B.J |  | hypothetical protein | -2.44 | 0 | 1 |
| F21F8.4 | *F21F8.4* | protease | -2.43 | 2 | 6 |
| AU113026 | *ttr-24* | domain | -2.40 | 5 | 1 |
| F54D5.3 |  | hypothetical protein | -2.38 | 0 | 2 |
| AV181428 |  | ATP transferase | -2.34 | 1 | 0 |
| AU113659 | *dod-19* | Downstream Of DAF-16 | -2.32 | 1 | 3 |
| C06B3.3 | *cyp-35C1* | CYtochrome P450 family | -2.31 | 1 | 1 |
| Y66A7A.6 | *gly-8* | GLYcosylation related | -2.30 | 0 | 0 |
| CEK120C8R | *mlc-1* | Myosin Light Chain | -2.29 | 3 | 1 |
| C14C6.2 |  | hypothetical protein | -2.28 | 3 | 1 |
| F55A12.1 |  | hypothetical protein | -2.27 | 1 | 1 |
| VZK822L.1 | *fat-6* | FATty acid desaturase | -2.26 | 1 | 2 |
| C50E3.13 |  | hypothetical protein | -2.25 | 3 | 7 |
| CEK111C2F | *ttr-41* | TransThyretin-Related family | -2.25 | 4 | 0 |
| F01G10.7 | *lipl-7* | hypothetical protein | -2.25 | 2 | 1 |
| T20D3.2 |  | hypothetical protein | -2.24 | 1 | 0 |
| F42F12.6 | *nspc-13* | Nematode Specific Peptide family | -2.23 | 1 | 1 |
| Y4C6A.3 |  | zinc finger protein | -2.21 | 0 | 0 |
| F02H6.2 |  | hypothetical protein | -2.21 | 1 | 0 |
| H12C20.2A | *pms-2* | PMS (Post Meiotic Segregation) | -2.21 | 5 | 0 |
| ZC308.4 |  | hypothetical protein | -2.20 | 3 | 5 |
| C16H3.2 | *lec-9* | gaLECtin | -2.19 | 2 | 2 |
| T14B4.4 | *tsp-10* | TetraSPanin family | -2.19 | 2 | 1 |
| F54F11.2 |  | hypothetical protein | -2.19 | 1 | 3 |
| C28C12.6 | *spp-10* | SaPosin-like Protein family | -2.18 | 2 | 1 |
| C01G5.8 | *fan-1* | hypothetical protein | -2.17 | 2 | 0 |
| T16H12.4 |  | hypothetical protein | -2.10 | 1 | 1 |
| F41D9.2 |  | hypothetical protein | -2.09 | 4 | 1 |
| T08A9.10 | *spp-6* | SaPosin-like Protein family | -2.07 | 2 | 2 |
| F19C7.1 |  | hypothetical protein | -2.06 | 0 | 3 |
| Y32B12A.3 |  | hypothetical protein | -2.06 | 2 | 2 |
| F32A7.7 | *str-245* | Seven TM Receptor | -2.05 | 1 | 1 |
| K08E3.3B | *toca-2* | TOCA (Transducer Of Cdc42-dependent Actin assembly) | -2.05 | 0 | 0 |
| AU116367 | *twk-18* | TWiK family of potassium channels | -2.01 | 4 | 0 |
| F42F12.1 | *nspc-9* | Nematode Specific Peptide family | -2.01 | 3 | 1 |

**Table S2.** List of genes downregulated on Rifampicin treatment determined by Microarray analysis along with the number of DAF-16 binding sites in their 5 kb upstream region
