# Supplementary material for: Rifampicin reduces advanced glycation end products and activates DAF-16 to increase lifespan in Caenorhabditis elegans
Source: Aging Cell. 2015 Feb 26;14(3):463–73. doi: 10.1111/acel.12327 (PMC4406675; doi:10.1111/acel.12327)
Supplement: Supplementary file 1 [file acel0014-0463-sd1.zip › Golegaonkar_Table S1_07012015.doc]

|  |  |  | **Fold**  **change** | **DAF-16 binding site motifs** | |
| --- | --- | --- | --- | --- | --- |
| **Sequence Name** | **Gene Name** | **Annotated function** |  | **Canonical** | **DAE** |
|  |  |  |  | **GTAAAC/TA** | **CTTATCA** |
| T16G1.6 |  | hypothetical protein | 80.61 | 5 | 1 |
| E02C1 |  | hypothetical protein | 76.63 | 2 | 0 |
| T16G1.4 |  | hypothetical protein | 23.97 | 1 | 2 |
| T16G1.7 |  | hypothetical protein | 22.50 | 1 | 1 |
| R05D8.8 | *dhs-14* | DeHydrogenases | 14.07 | 1 | 1 |
| F09G2.6 | *ugt-36* | UDP-GlucuronosylTransferase | 13.84 | 2 | 3 |
| C47A10.1 | *pgp-9* | P-GlycoProtein related | 11.07 | 1 | 4 |
| B0213.14 | *cyp-34A8* | CYtochrome P450 family | 8.79 | 2 | 2 |
| Y38E10A.14 |  | hypothetical protein | 7.97 | 3 | 3 |
| C45H4.2 | *cyp-33* | CYtochrome P450 family | 6.82 | 2 | 1 |
| ZK596.1 |  | hypothetical protein | 5.72 | 3 | 2 |
| F41B5.2 | *cyp-33C7* | CYtochrome P450 family | 5.60 | 1 | 2 |
| R08F11.3 | *cyp-33C8* | CYtochrome P450 family | 5.51 | 0 | 1 |
| B0213.15 | *cyp-34A9* | CYtochrome P450 family | 4.60 | 1 | 2 |
| T28A11.16 |  | hypothetical protein | 4.44 | 4 | 0 |
| F13D11.4 |  | dihydroflavonol-4-reductase | 4.37 | 6 | 2 |
| F47B8.10 |  | hypothetical protein | 4.17 | 2 | 1 |
| B0285.9 | *ckb-2* | Choline Kinase B | 3.90 | 1 | 0 |
| M88.1 | *ugt-62* | UDP-GlucuronosylTransferase | 3.86 | 2 | 3 |
| T28A11.19 |  | hypothetical protein | 3.74 | 0 | 1 |
| T10B9.10 | *cyp-13A7* | CYtochrome P450 family | 3.74 | 3 | 2 |
| T23F4.2 |  | hypothetical protein | 3.56 | 0 | 3 |
| R09B5.11 |  | sugar transporter | 3.56 | 0 | 3 |
| C01B4.7 |  | hypothetical protein | 3.43 | 1 | 1 |
| F59C12.4 |  | hypothetical protein | 3.37 | 1 | 0 |
| C25F9.6 |  | hypothetical protein | 3.29 | 1 | 1 |
| C33A12.6 | *ugt-21* | UDP-GlucuronosylTransferase | 3.21 | 3 | 3 |
| T20D4.1 |  | hypothetical protein | 3.17 | 1 | 1 |
| Y19D10A.4 |  | hypothetical protein | 3.14 | 1 | 1 |
| ZK930.2 |  | hypothetical protein | 3.14 | 0 | 0 |
| F16F9 |  | lipase | 3.11 | 2 | 0 |
| B0331.2 |  | phosphate permease like | 3.03 | 4 | 0 |
| F52E1.8 | *pho-6* | hypothetical protein | 3.02 | 3 | 0 |
| F20G2.1 |  | dehydrogenase | 3.00 | 1 | 0 |
| F12E12.11 |  | hypothetical protein | 2.95 | 1 | 0 |
| F59F5.2 | *glo-3* | hypothetical protein | 2.88 | 3 | 5 |
| C04H5.2 | *clec-147* | C-type LECtin | 2.88 | 1 | 3 |
| C08B6.1 | *ugt-17* | UDP-GlucuronosylTransferase | 2.88 | 2 | 1 |
| ZK669 |  | hypothetical protein | 2.87 | 3 | 1 |
| D1054.8 |  | dehydrogenase | 2.86 | 2 | 3 |
| C06E4.3 |  | dehydrogenase | 2.82 | 2 | 0 |
| F55H2.1 | *sod-4* | SOD (superoxide dismutase) | 2.82 | 2 | 1 |
| Y55B1BL.1 |  | hypothetical protein | 2.77 | 2 | 1 |
| R05D7.5 |  | hypothetical protein | 2.74 | 0 | 0 |
| R12H7.3 | *skr-19* | SKp1 Related (ubiquitin ligase) | 2.63 | 2 | 2 |
| C37H5.6 |  | hypothetical protein | 2.62 | 1 | 0 |
| F59B10.4 |  | hypothetical protein | 2.55 | 2 | 0 |
| ZK813.2 |  | hypothetical protein | 2.52 | 1 | 0 |
| F32G8.6 | *cat-4* | abnormal CATecholamine | 2.48 | 2 | 2 |
| T22C8.8 | *vab-9* | Variable ABnormal morphology | 2.44 | 0 | 0 |
| T24H10.7 | *jun-1* | JUN transcription factor | 2.41 | 3 | 1 |
| Y41C4A.13 |  | hypothetical protein | 2.41 | 0 | 1 |
| Y54G2A.O | *ubc-13* | UBiquitin Conjugating enzyme | 2.37 | 4 | 0 |
| T04C9.4A | *mlp-1* | MLP/CRP family (Muscle LIM Protein/Cysteine-rich Protein) | 2.34 | 4 | 0 |
| T19D2.2 | *prl-1* | hypothetical protein | 2.33 | 1 | 4 |
| F16B4.2 | *F16B4* | hypothetical protein | 2.33 | 1 | 2 |
| F55A12.7 | *apm-1* | AdaPtin, Mu/medium chain | 2.29 | 1 | 2 |
| C53D5.2 |  | hypothetical protein | 2.27 | 1 | 0 |
| C47D12.5 |  | hypothetical protein | 2.27 | 4 | 2 |
| W10G6.2 | *sgk-1* | Serum- and Glucocorticoid- inducible kinase homolog | 2.26 | 4 | 2 |
| K09A9.2 | *rab-14* | RAB family | 2.24 | 3 | 0 |
| F13H6.1 |  | hypothetical protein | 2.23 | 1 | 1 |
| F36A2.14 |  | hypothetical protein | 2.21 | 2 | 0 |
| F35C11.4 |  | hypothetical protein | 2.21 | 4 | 3 |
| K02E2.6 |  | hypothetical protein | 2.20 | 2 | 0 |
| T07A9.9 |  | hypothetical protein | 2.20 | 1 | 0 |
| C01G10.5 |  | hypothetical protein | 2.18 | 3 | 0 |
| K10B4.6 | *cwn-1* | WNT family | 2.18 | 1 | 0 |
| F26A3.1 |  | hypothetical protein | 2.17 | 1 | 0 |
| C08C3.3 | *mab-5* | Homeodomain transcription factor | 2.17 | 2 | 3 |
| Y37D8A.16 |  | hypothetical protein | 2.16 | 3 | 0 |
| R74.3 | *xbp-1* | X-box Binding Protein homolog | 2.16 | 3 | 1 |
| W06E11.4 | *sbds-1* | Shwachman-Bodian-Diamond Syndrome protein homolog | 2.16 | 1 | 0 |
| R07H5.4 | *sdz-27* | SKN-1 Dependent Zygotic transcript | 2.15 | 4 | 1 |
| K10D6.4 |  | hypothetical protein | 2.14 | 1 | 0 |
| H06H21.10 | *tat-2* | Transbilayer Amphipath Transporters (subfamily IV P-type ATPase) | 2.13 | 3 | 3 |
| F54E7.7 | *rcn-1* | Regulator of CalciNeurin | 2.13 | 4 | 1 |
| F58B6.2 | *inft-1* | INverted Formin/formin Three-related /// locus:inft-1 | 2.11 | 2 | 1 |
| T28D6.2 | *tba-7* | TuBulin, Alpha /// locus:tba-7 | 2.11 | 1 | 1 |
| F44G3.8 | *fbxa-143* | F-box A protein | 2.10 | 2 | 4 |
| F14D2.7 |  | hypothetical protein | 2.10 | 0 | 0 |
| C45G9 |  | hypothetical protein | 2.10 | 2 | 0 |
| T19C3.4 |  | Polyposis locus protein 1 | 2.10 | 4 | 1 |
| F58G6.1 | *amph-1* | AMPHiphysin homolog | 2.09 | 3 | 1 |
| K03E6.1 | *lim-6* | LIM domain family | 2.08 | 2 | 2 |
| K03E6.3 | *ncs-3* | Neuronal Calcium Sensor family | 2.08 | 1 | 0 |
| T27A1.5 |  | hypothetical protein | 2.07 | 2 | 4 |
| C04C3.4 |  | hypothetical protein | 2.07 | 4 | 0 |
| Y47D9A.2 | *scpl-3* | SCP(Small C-terminal domain Phosphatase)-Like phosphatase | 2.07 | 0 | 0 |
| AV177366 | *nhr-97* | Nuclear Hormone Receptor family | 2.07 | 3 | 2 |
| C14F11.3 | *lite-1* | high-energy LIghT unrEsponsive | 2.06 | 2 | 0 |
| K09C8.1 | *pbo-4* | PBOc defective (defecation) | 2.05 | 1 | 1 |
| H09F14.1 |  | 7 transmembrane receptor (rhodopsin family) | 2.05 | 0 | 0 |
| F53F10.4 | *unc-108* | UNCoordinated | 2.03 | 3 | 2 |
| Y23H5A.6 | *ctn-1* | alpha-CaTuliN (catenin/vinculin related) | 2.03 | 0 | 1 |
| Y77E11A.2 |  | hypothetical protein | 2.03 | 4 | 3 |
| W02D9.7 |  | hypothetical protein | 2.03 | 1 | 2 |
| F54C9.1 | *iff-2* | Initiation Factor Five (eIF-5A) homologs | 2.01 | 3 | 0 |
| K10B4.3 |  | hypothetical protein | 2.01 | 2 | 1 |
| F16B4.9 | *nhr-178* | Nuclear Hormone Receptor family | 2.01 | 0 | 0 |
| Y37H2C.1 |  | hypothetical protein | 2.01 | 0 | 0 |
| C05D2.8 |  | hypothetical protein | 2.00 | 2 | 3 |

**Table S1.** List of genes upregulated on Rifampicin treatment determined by Microarray analysis along with the number of DAF-16 binding sites in their 5 kb upstream region
