# Supplementary material for: Rifampicin reduces advanced glycation end products and activates DAF-16 to increase lifespan in Caenorhabditis elegans
Source: Aging Cell. 2015 Feb 26;14(3):463–73. doi: 10.1111/acel.12327 (PMC4406675; doi:10.1111/acel.12327)
Supplement: Supplementary file 1 [file acel0014-0463-sd1.zip › Golegaonkar_Supporting material_010115.doc]

**Supplementary Materials and methods**

**Strains used in the study**

Unless otherwise mentioned, all strains were maintained at 20 ^o^C using standard *C. elegans* techniques (Stiernagle 2006). Strains used in the study are: N2 Bristol as wild-type, *daf-16(mgDf50)I, glod-4(gk189)III, daf-18(e1375)IV, pdk-1(mg142)X, akt-1(mg144)V, CL2006- dvIs2[pCL12(unc-54/human Abeta peptide 1-42 minigene) + pRF4], jnk-1(gk7)IV* and *jkk-1(km2)X*.

**Determination of IC50:**

IC_50_ of glycation inhibitors was determined by MALDI-ToF-MS based insulin glycation assay (Golegaonk*ar et a*l. 2010). 2 M stock glucose (Sigma, USA) and 2 mg/ml insulin (Sigma, USA) was prepared in 0.1 M phosphate buffer pH 7.4. Each glycation reaction was performed with 50 µL of insulin, 25 µL of glucose and 25 µL of Rifampicin, Rifamysin SV or Rifaximin, Thus, the final reaction concentration of insulin was 0.00001724 mM, 0.5 M glucose and RIF, RSV and RMN concentration was in the range of 0.0078 mM to 0.5 mM. The reaction was incubated at 37˚C for seven days. Mass spectral acquisition were done by mixing the reaction mixture with sinapinic acid (10 mg of sinapinic acid prepared in 30/70 ACN: water with 0.1% TFA), in 1:10 ratio. The samples were spotted on a stainless steel plate in triplicate and allowed to dry at room temperature. Insulin glycation was monitored by MALDI-ToF/ToF-MS (ABSCIEX 5800, MALDI-ToF/ToF) equipped with a Opti-Beam™ On-axis Nd:YAG. The spectra were acquired in the mass range of 4000 to 7000 m/z in a positive linear mode with the following acquisition settings. The acceleration voltage was set to 12 kV, delayed ion extraction time of 500 ns and the low mass ion gate was set to 500 Da. A fixed laser intensity of 4400 was used for all spectral acquisitions. Each spectrum was acquired for 5 times with 250 shots per spectrum in 3 different technical experiments. The spectra were processed for advanced baseline correction, noise removal and mass calibration. The per cent glycation inhibition was calculated by monitoring the relative intensity of glycated insulin. The apparent IC_50_ was calculated using the formula (C–T) /C × 100, where C is the relative intensity of glycated insulin in the absence of an inhibitor and T is the relative intensity of glycated insulin in the presence of an inhibitor.

**Life span analysis**

*E. coli* OP50 transformed with pL4440 (OP50-Amp) was grown at 37 ^o^C for 14 hrs in LB broth (having 100 mg/L ampicillin). The bacterial growth medium was centrifuged and the bacterial pellet was resuspended in S-complete at a concentration of 100 mg/ml. S-complete media (Stiernagle 2006) was prepared by mixing S-Basal with 10 ml of 1M Potassium citrate buffer pH 6.0, 10 ml of trace metal solution (1.86 g disodium EDTA, 0.69 g FeSO_4_ heptahydrate, 0.2 g MnCl_2_ tetrahydrate, 0.29 g ZnSO_4_ heptahydrate, 0.025 g CuSO_4_ pentahydrate in 1L of water), 3 ml each of 1M CaCl_2_ dihydrate and MgSO_4_ heptahydrate, 0.5 ml cholesterol (from stock of 10 mg/ml in ethanol) and filter sterilized using 0.2 μ filter. The bacterial suspension was heat-killed at 100 ^o^C for 45 min and stocks kept at 4 ^o^C till use.

Gravid adult worms were bleached and eggs placed on OP50-seeded Nematode Growth Media (NGM) plates. The worms were allowed to grow at 20^o^C till young adult stage, rinsed off the plates and washed 4 times with 1X M9 buffer (Stiernagle 2006). Worms were pelleted at 2000 rpm for 1 min and resuspended in S-complete and suspension was diluted further to contain 8-12 worms per 10 μl.

Liquid life span was performed in Non-coated sterile 96-well plates (Corning, USA). A master-mix was prepared such that each well of the 96-well culture plates contained 80 μl of S-complete, 10 μl of resuspended worms (8-12 worms), 9.9 μl of heat-killed OP50-Amp bacteria (100 mg/ml stock), 1.6 μl of carbenicillin (50 mg/ml stock) and 0.6 μl Amphoterecin B (250 μg/ml stock; Sigma, USA). To each well 30 μl of 0.6 mM FuDr was added. The next day, 10 μl of a solution in S-complete buffer containing 50 μM of either RIF, RSV or RMN (from 100 mM stock in DMSO) or 0.2% of DMSO was added and volume made up to 150 μl by further adding 10 μl of S-complete. The worms were fed again with 9 ul of 100 mg/ml heat-killed OP50-Amp per well on 5^th^ day. Day of drugs addition is considered as Day 1. Every other day, the worms in the wells were exposed to light and plates gently tapped to agitate the worms in solution and based on their movement were scored as dead or alive. Dead worms were not extracted from the well; a count of total number of live worms in each well was kept to plot the life span. Multiple wells (at least 6) were used for each concentration of drug. Every plate had the relevant no drug controls. Life span data from multiple experiments were pooled to generate graph, details of which are available in Table S3.

**Statistical Analysis**

Statistical analyses for survival were conducted using Mantel-Cox log rank test with OASIS software available at http://sbi.postech.ac.kr/oasis (Ya*ng et a*l. 2011). *P* value calculations for other experiments were performed by Unpaired T test using SigmaPlot 10.0 (Systat software, USA).

**Proteotoxicity**

Gravid adult CL2006 (Link 1995) transgenic worms were bleached and eggs hatched at 15 ^o^C on live OP50-containing NGM agar plates. Assay was set up at 20 ^o^C in the same way as discussed above. Worms from three wells were transferred to an OP50-seeded NGM agar plate and scored for paralysis by prodding with a platinum wire. Worms showing whole body movement were considered alive and those that only moved their head on prodding were considered paralysed.

**DAF-16 translocation**

The *daf-16:gfp* translational fusion strain (Henderson & Johnson 2001) was grown on NGM plates at 15 ^o^C. The gravid adult worms were bleached and ~100 eggs were transferred to NGM plates supplemented with either 0.35% DMSO or 500 μM RIF, RSV or RMN plates. The worms were grown till L3 and mounted on 2% agarose slides using 10 mM sodium azide. The GFP was categorized as nuclear, cytoplasmic or intermediate depending on the extent of DAF-16 translocation into the nucleus as visualized using a fluorescent microscope (Axioimager M2, Zeiss, Germany). In these experiments, worms were not subjected to heat shock.

**RNA isolation**

Worms grown in liquid culture as above were washed thrice in 1X M9 buffer and 0.3 ml of Trizol reagent (Invitrogen, USA) was added to the worm pellet. The worms were lysed by vigorous vortexing and RNA purified by ethanol precipitation. The concentration and the purity of the RNA were determined using Bioanalyzer (Agilent, USA). Alternatively, the quality of the ribosomal 28 S and 18 S as visualized on an agarose gel was used as a measure of integrity. Absorbance at 260/280 nm was used to determine quantity.

**Microarray and QRT-PCR analysis**

Microarray analysis was carried out commercially at Ilife Discoveries (Gurgaon, India) using an Affymetrix, USA platform. Briefly, biotinylated cRNA were prepared according to the standard Affymetrix protocol from 6 ug total RNA of Wild-type treated with DMSO or RIF in two biological replicates (Expression Analysis Technical Manual, 2001, Affymetrix). Following fragmentation, cRNA were hybridized for 16 hr at 45 ^o^C on GeneChip *C.elegans* Genome Array. GeneChips were washed and stained in the Affymetrix Fluidics Station 450. GeneChips were scanned using the Hewlett-Packard GeneArray Scanner 3000 7G. The primary data was analyzed with Affymetrix Expression Console. Further differential gene expression was performed using Genespring GX 11.1 with Affymetrix default analysis settings and RMA was used for normalization. The probes of intensities less than 20 percentile in the raw data were excluded prior to differential gene expression analysis. More than two fold expression changes with *p* value <0.05 (student's *t* test) are reported. The detailed microarray data, with accession number GSE45292 is available at <http://www.ncbi.nlm.nih.gov/geo/query/acc.cgi?token=xjcdjagswaygerq&acc=GSE45292>. For Gene Ontology term analysis reported in the text, a cutoff of 1.5 folds and *p* < 0.05 were taken and DAVID software (Denn*is et a*l. 2003) used.

cDNA was synthesized from two independent biological replicates using 5 µg of total RNA using the SuperScript III cDNA synthesis kit (Invitrogen, USA). Expression levels of transcripts were determined by quantitative real time PCR (QRT-PCR) using the Mesagreen MasterMix (Eurogentec, Belgium). Relative gene expression, normalized to actin, was determined by the comparative C_T_ method (Schmittgen & Livak 2008), between worms treated with DMSO and RIF.

**Protein extraction**

In a 250 ml flask, 17.78 ml S-complete, 2.20 ml young-adult worm suspension (5000 worms), 1.98 ml heat-killed OP50-Amp (100 mg/ml stock), 33 μl of Carbenicillin (50mg/ml stock; Sigma, USA), 13.2 μl of Amphoterecin B (250 μg/ml stock; Sigma, USA) and 6.6 ml of 0.6 mM FuDr were added. The next day, 2.2 ml of S-complete containing 200 µM RIF or RMN (from a 100mM stock made in DMSO) or 0.2% DMSO (final concentrations) was added. The culture was grown for the required number of days as indicated in the figures (fed once again on day 5 with 1.98 ml OP50-Amp) at 20 ^o^C and harvested. The pellet, obtained by centifugation at 1000 rpm for 1 min, was washed four times with M9 buffer and flash frozen. The pellet was freeze-thawed 4 times in protein extraction buffer (20 mM Hepes buffer pH 7.9, 25% glycerol, 0.42 mM NaCl, 1.5 mM MgCl_2_ hexahydrate , 0.2 mM EDTA dihydrate, 0.5 mM DTT) with protease inhibitor cocktail (Sigma,USA), sonicated and pelleted at 10,000 rpm 10 mins. The protein concentration in the supernatant was estimated by using Bradford reagent (BioRad, USA).

**Western blotting:**

About 30 µg of protein was separated on 12% SDS-PAGE and transferred to PVDF membrane. The membrane was blocked for 1 hr at room temperature with 5% non-fat milk. The blot was incubated overnight at 4^˚^C with anti-CML monoclonal antibodies (Catalog No. KAL-KH024, Cosmo Bio Co Ltd., Japan) at 1:1000 dilution in 2.5% non-fat milk. Later, the membrane was washed thrice with TBST (TBS with 0.1% Tween 20) and further incubated in 1:10000-diluted secondary antibody (anti-mouse conjugated to HRP) for 1 hr at room temperature. The blot was then washed four times with TBST, each wash lasting 10 min. The blot was developed using enhanced chemiluminiscent substrate (Millipore, USA). In case of α-Tubulin (Catalog No. 2125S) or β-Actin (Catalog No. 4970S) (Cell Signaling Technology, USA;) western blots, primary antibody (1:1000) was made in 5% BSA and incubated overnight at 4˚C. The membrane was washed thrice with TBST and secondary antibody (anti-rabbit conjugated to HRP) incubation was performed for 1 hr at room temperature.

**LC-MS^E^, Protein Identification, Database Search, and PTM Analysis**

Prior to LC-MS^E^ analysis, 100 µg of protein was washed with 0.1 % Rapigest and digested with trypsin after reduction and alkylation with 100 mM DTT and 200 mM iodoacetamide, respectively. Two microliters of digested peptides (100 ng/μL ) was spiked with 100 femtomoles of digested yeast enolase as an internal standard were analyzed using online nanoACQUITY UPLC coupled to a SYNAPT HDMS (Waters Corporation, Milford, MA) by MS^E^, a label free relative quantification approach . After MS^E^ analysis, data was analyzed with Protein Lynx Global Server software (PLGS version 2.5 Waters Corporation, Milford, MA). A preliminary search was performed involving carbamidomethylation of cyteine residues and oxidation of methionine as fixed and variable modifications, for protein identification against the UniProt *C. elegans* database. Data was acquired in triplicates and the commonly identified proteins were analyzed for fold change (Table S4). Glycation modification including Amadori, Carboxy methyl lysine (CML), Carboxy ethyl lysine (CEL), Pyraline, Glyoxalderivedhydroimidazolone (GH1), Argpyrimidine (ARGPYR), Methylglyoxal-derived hydroimidazolone (MG-H1).were identified by subjecting PLGS search against subset of protein databases that were identified in the preliminary search as described earlier (Bhons*le et a*l. 2012; Banso*de et a*l. 2013). The false positive rate was set to 1% in the PLGS workflow. The PLGS-identified glycated peptides were manually validated using the following criteria: (a) all the modified peptides should be present in at least duplicates (b) have correct parent ion mass shift values, (c) presence of precursor ion in the MS/MS spectra ensured matched fragment ions are asscociated with its precursor (d) glycated peptides should have both b and y ions in the MS/MS spectra (d) glycated peptides should have a minimum of seven fragment ions matching with the sequence. (e) CML modified peptides were analyzed for the presence of diagnostic reporter ions (Graifenhag*en et a*l. 2014). The PLGS identified glycation modifications not following above mentioned criteria were not considered in this study.

**Supplementary Figure legends**

**Figure S1:** (A) Pathways by which AGEs are formed in animal cells, as collated from Singh *et al.* 2001, Ulrich & Cerami 2001 and Ahmed 2005. (B) Mass spectrometry traces for the *in vitro* glycation experiments. The spectra show insulin mass of 5808 Da (indicated using #) as well as glycated insulin mass of 5970 Da (indicated by arrows). The glycated insulin mass in presence or absence of RIF, RSV or RMN were monitored.

**Figure S2:** (A) RIF increased life span on live *E. coli* OP50 in a concentration dependent manner. (B) Kanamycin (KAN) had little effect on life span in WT worms. (C) RIF treatment suppressed gut autofluorescence in WT worms as visualized on Day 12. (D) RIF treatment reduced Aβ_1-42_-induced paralysis in CL2006 transgenic strain. Paralysis was scored on different days post-L4 larval stage, as mentioned in the x axis. Error bars indicate SEM; *p* value (student's *t* test) of difference between DMSO and RIF treatment is shown.

**Figure S3:** *(A)* The life span of *glod-4* mutant allele, *glod-4(gk189,)* is significantly shorter than WT. *(B)* RIF reduced CML levels in *glod-4(gk189)* as detected by western blot analysis using α-CML antibody. The α-Tubulin (α-TUB) levels were used for normalization. Quantification of the blot is provided on the right. *(C)* RIF significantly increased life span of *glod-4(gk189*).

**Figure S4:** RIF increased life span of activated PDK-1 strain, *pdk-1(mg142).*

**Figure S5:** *(A)* GO term analysis of genes that were significantly downregulated upon RIF treatment. *(B)* Quantitative real-time PCR assay revealed that 7 out of 10 genes that were investigated changed expression in a *daf-16*-dependent manner when treated with RIF.

**Supplementary Table legends**

Table S1. List of genes upregulated on Rifampicin treatment determined by Microarray analysis along with the number of DAF-16 binding sites in their 5 kb upstream region

Table S2. List of genes downregulated on Rifampicin treatment determined by Microarray analysis along with the number of DAF-16 binding sites in their 5 kb upstream region

Table S3: Details of life spans that are reported in the manuscript

Table S4: Relative quantification of fold changes of proteins detected by LC-MS^E^

**References:**

Bansode SB, Chougale AD, Joshi RS, Giri AP, Bodhankar SL, Harsulkar AM , Kulkarni MJ (2013). Proteomic analysis of protease resistant proteins in the diabetic rat kidney. *Mol Cell Proteomics*. **12**, 228-236.

Bhonsle HS, Korwar AM, Kote SS, Golegaonkar SB, Chougale AD, Shaik ML, Dhande NL, Giri AP, Shelgikar KM, Boppana R , Kulkarni MJ (2012). Low plasma albumin levels are associated with increased plasma protein glycation and HbA1c in diabetes. *J Proteome Res*. **11**, 1391-1396.

Dennis G, Jr., Sherman BT, Hosack DA, Yang J, Gao W, Lane HC , Lempicki RA (2003). DAVID: Database for Annotation, Visualization, and Integrated Discovery. *Genome Biol*. **4**, P3.

Golegaonkar SB, Bhonsle HS, Boppana R , Kulkarni MJ (2010). Discovery of rifampicin as a new anti-glycating compound by matrix-assisted laser desorption/ionization mass spectrometry-based insulin glycation assay. *Eur J Mass Spectrom (Chichester, Eng)*. **16**, 221-226.

Graifenhagen U, Nguyen VD, Moschner J, Giannis A, Frolov A , Hoffmann R (2014). Sensitive and site-specific identification of carboxymethylated and carboxyethylated peptides in tryptic digests of proteins and human plasma. *J Proteome Res*.

Henderson ST , Johnson TE (2001). daf-16 integrates developmental and environmental inputs to mediate aging in the nematode Caenorhabditis elegans. *Curr Biol*. **11**, 1975-1980.

Link CD (1995). Expression of human beta-amyloid peptide in transgenic Caenorhabditis elegans. *Proc Natl Acad Sci U S A*. **92**, 9368-9372.

Schmittgen TD , Livak KJ (2008). Analyzing real-time PCR data by the comparative C(T) method. *Nature protocols*. **3**, 1101-1108.

Stiernagle T (2006). Maintenance of C. elegans. *WormBook*, 1-11.

Yang JS, Nam HJ, Seo M, Han SK, Choi Y, Nam HG, Lee SJ , Kim S (2011). OASIS: online application for the survival analysis of lifespan assays performed in aging research. *PLoS One*. **6**, e23525.
